# Identification of Implications of Angiogenesis and m6A Modification on Immunosuppression and Therapeutic Sensitivity in Low-Grade Glioma by Network Computational Analysis of Subtypes and Signatures

**DOI:** 10.3389/fimmu.2022.871564

**Published:** 2022-04-27

**Authors:** Bo Li, Fang Wang, Nan Wang, Kuiyuan Hou, Jianyang Du

**Affiliations:** ^1^ Department of Neurosurgery, Huangyan Hospital, Wenzhou Medical University, Taizhou, China; ^2^ Department of Neurosurgery, Taizhou First People’s Hospital, Taizhou, China; ^3^ Department of Neurosurgery, The Second Affiliated Hospital of Harbin Medical University, Harbin, China; ^4^ Department of Neurosurgery, The First Hospital of Qiqihar City, Qiqihar, China; ^5^ Department of Neurosurgery, Shandong Provincial Hospital Affiliated to Shandong First Medical University, Jinan, China

**Keywords:** m6A RNA modification, angiogenesis, tumor immune microenvironment, immunosuppression, low-grade glioma

## Abstract

Angiogenesis is a complex process in the immunosuppressed low-grade gliomas (LGG) microenvironment and is regulated by multiple factors. N^6^-methyladenosine (m6A), modified by the m6A modification regulators (“writers” “readers” and “erasers”), can drive LGG formation. In the hypoxic environment of intracranial tumor immune microenvironment (TIME), m6A modifications in glioma stem cells are predominantly distributed around neovascularization and synergize with complex perivascular pathological ecology to mediate the immunosuppressive phenotype of TIME. The exact mechanism of this phenomenon remains unknown. Herein, we elucidated the relevance of the angiogenesis-related genes (ARGs) and m6A regulators (MAGs) and their influencing mechanism from a macro perspective. Based on the expression pattern of MAGs, we divided patients with LGG into two robust categories *via* consensus clustering, and further annotated the malignant related mechanisms and corresponding targeted agents. The two subgroups (CL1, CL2) demonstrated a significant correlation with prognosis and clinical-pathology features. Moreover, WGCNA has also uncovered the hub genes and related mechanisms of MAGs affecting clinical characters. Clustering analysis revealed a synergistic promoting effect of M6A and angiogenesis on immunosuppression. Based on the expression patterns of MAGs, we established a high-performance gene-signature (MASig). MASig revealed somatic mutational mechanisms by which MAGs affect the sensitivity to treatment in LGG patients. In conclusion, the MAGs were critical participants in the malignant process of LGG, with a vital potential in the prognosis stratification, prediction of outcome, and therapeutic sensitivity of LGG. Findings based on these strategies may facilitate the development of objective diagnosis and treatment systems to quantify patient survival and other outcomes, and in some cases, to identify potential unexplored targeted therapies.

## Introduction

Low-grade gliomas (LGG, World Health Organization, WHO II and III) consist of diffuse low- and moderate-grade gliomas (1). Unlike glioblastoma (GBM, WHO IV), with a 5-year survival rate of only 5.6%, LGG has a median survival period of more than seven years and is relatively less invasive (2). GBM and LGG possessed considerable heterogeneity in pathological characteristics and clinical results (3). Traditionally, patients with grade II gliomas harbored a better prognosis than that of grade III. Still, since the WHO redefined the grade of gliomas in 2016, changes in molecular pathology have been considered (which can be objectively determined) more important than this grade level. Excitingly, a set of genetic features (which is something subjective and tumor tissue-dependent) that are specific to LGG and are closely related to histological and clinical outcomes were identified (4). These genetic markers mainly include high-frequency mutations in genes encoding isocitrate dehydrogenase (IDH) 1 and 2, and codeletion of chromosomal arms 1p and 19q (1p/19q) (5, 6). The TCGA Cancer Research Center proposes to divide LGG into IDH wild-type (IDHwt), and additionally IDH mutants (IDHmut-non-codel) with euploid 1p/19q and IDH mutants (IDHmut-codel) with 1p/19q deletion (4, 7, 8). Although surgical resection, postoperative radiotherapy, and chemotherapy can prolong the survival time of patients, the recurrence outcome is inevitable. Despite the specific treatment for the above classification, the prognosis of LGG is still not significantly improved. Therefore, selected key gene sets for stratification of LGG patients and construction of tumor prediction models could provide new strategies for more precise molecular subtyping and corresponding personalized treatment.

The role of RNA in tumor processes has gradually gained attention in the past decade and has quickly become a focus area for cancer research (9–12). Adenosine N^6^-methyladenosine (m6A) was considered to be the most common internal modification (10). The accumulation of m6A modification was regulated by methyltransferases (“writers”), including methyltransferase like 3/14 proteins (METTL3/14) (13, 14) and cofactors WTAP (15), RBM15/15B (16), KIAA1429 (VIRMA) (17), and ZC3H13 (18). The realization of m6A biology function depends on the “readers”, include YTHDC1/2 (19), YTHDF1/2/3 (20), HNRNPA2B1 (21), IGF2BP1 (22) and EIF3A (23). As the function of specifically removing the methyl from modified mRNAs, “erasers” mainly include FTO (24, 25) and ALKBH5 (26). Starting with the identification of RNA demethylases and the establishment of methylated RNA sequencing protocols, RNA methylation has become a common phenomenon and a key regulator of RNA transcription (27, 28), the event of processing (29, 30), splicing (31, 32), RNA stabilities (33, 34), and translation (23, 35). Notably, the functional role of m6A modification in cancer-related processes has also received increasing attention (36, 37). Since scholars only focus on the specific functions or specific cellular pathways of designated m6A regulators, most of the existing studies have apparent limitations. Therefore, an integrative analysis of the expression of m6A regulators in LGG was urgent needs to be explored.

Given the critical role of angiogenesis in LGG, the use of angiogenesis-related genes (ARGs) to provide valid risk stratification and identify potential regulatory mechanisms appear to be promising (38). Angiogenesis is an important process in the development of tumorigenesis. Tumor cells release several pro-angiogenic factors, such as vascular endothelial growth factor A (VEGFA), which promote neovascularization in the tumor immune microenvironment (TIME) (39). In the hypoxic environment of intracranial TIME, m6A modification of glioma stem cells and neovascularization promote each other to form an immunosuppressive phenotype of TIME (40–42). Thus, anti-angiogenic therapy and m6A site-targeted therapy have been shown to significantly improve the prognosis of LGG patients (43–45). A comprehensive multi-omics analysis of m6A regulators and ARGs (MAGs) may make a theoretical contribution to the prognosis of LGG, the immunosuppressive profile of TIME, and other features.

In this study, we focused on elucidating the correlation between m6A modification and angiogenesis and the mechanisms of their effects from a macroscopic perspective and constructing a high-performance prognostic survival gene signature based on expression patterns. m6A modification and angiogenesis-associated risk score and gene signature (MASig) were constructed based on the expression values, and we found that MASig performed well in survival prediction. The results of this study are expected to provide a more comprehensive genomic map of intracranial immunosuppression due to epigenetic modifications and angiogenesis and may lead to a better prognostic prediction strategy for human LGG.

## Materials and Methods

The materials and Methods section was arranged in the Supporting materials (**Supplementary Method**).

## Results

### Transcriptome and Proteome Levels of m6A Regulators and Clinical Parameters in LGG

**Figure 1:**
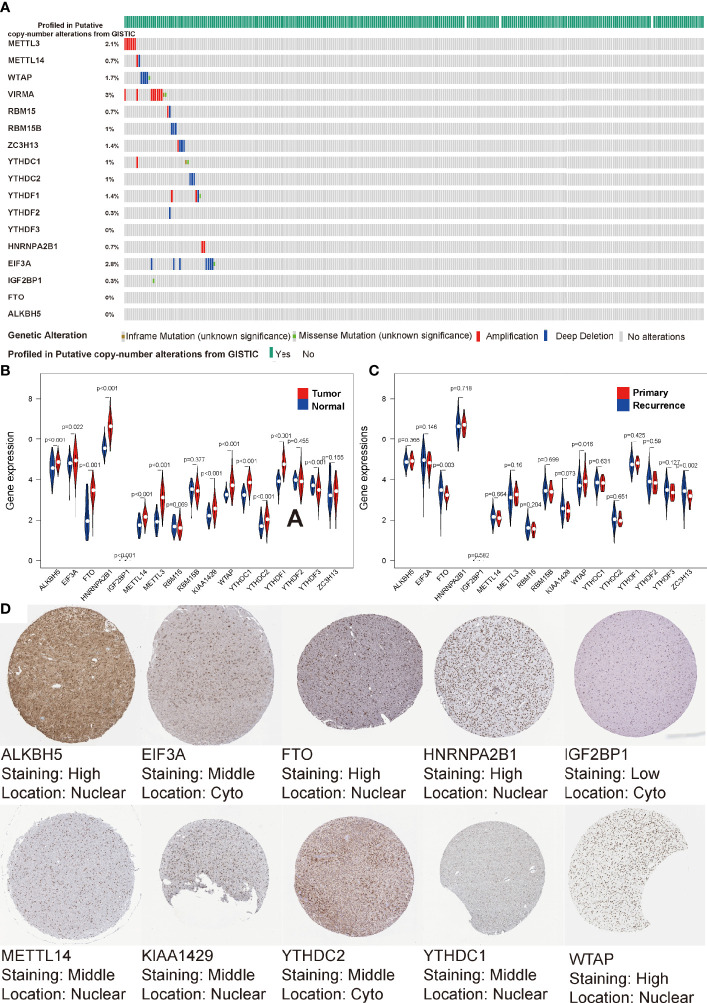
Transcriptome and proteome levels of m6A regulators and clinical parameters in low-grade gliomas (LGG). **(A)** Genetic alteration profiles of the studied m6A modification regulators from 529 patients with LGG. **(B, C)** Violin diagrams of expression levels of the m6A regulators (normal sample *vs* tumor sample) **(B)** and (primary LGG *vs* recurrent LGG) **(C)** from TCGA database. **(D)** Immunohistochemistry staining of m6A RNA regulators in LGG retrieved from Human Protein Atlas.

It has been reported that angiogenesis and hypoxia in glioma TIME lead to the accumulation of Glioma Stem Cells (GSC) around small vessels (41, 45). m6A, the most popular modification pattern of GSC mRNA, plays an important role in the treatment tolerance and stemness maintenance of GSC. To uncover the regulatory patterns among them, we first examined the mutational panorama of m6A regulators. Notably, we observed that the frequencies of genetic abnormal (coy number or mutation change) with the 17 regulators were quite low (ranging from 0% to 3%) in the LGG cohort, indicating the stability of their transcriptional levels when performing biological functions (**Figure 1A**). We also comprehensively analyzed the relationship between each m6A regulator and the type of sample (normal sample, primary LGG, relapsed LGG). We found that with the exception of RBM15/15B, YTHDF2, ZC3H13, all other regulators were significantly correlated with tumor occurrence (P < 0.05, **Figure 1B**). Furthermore, FTO, KIAA1429, WTAP, ZC3H13 were significantly associated with tumor recurrence (P < 0.05, **Figure 1C**). Moreover, the IHC profiles were obtained from HPA also illustrated the expression status of the m6A regulators, along with the corresponding location. As shown in **Figure 1D**, the proteome levels of most m6A regulators were consistent with their expression levels in transcription, but HPA does not contain any IHC details of YTHDF1, YTHDF2, YTHDF3, RBM15, RBM15B, ZC3H13 in the LGG cohort.

Copy number variation (CNV) and methylation are the molecular mechanisms by which genetic abnormalities lead to carcinogenesis. In the CNV difference analysis of normal and LGG, we found that there were significant differences in the five regulators, which were IGF2BP1, FTO, METTL3, YDHTC1, and YDHTC2 (**Figure S1A**). Among 17 regulators, we found that the expression values of ten regulators were significantly correlated with CNV **(** **Figures S1B–K** **)**. We found that double deletion was associated with low expression, and amplification was significantly associated with high expression. As shown in **Figure S1L**, we searched the methylation differences of these regulators from the DiseaseMeth database and found that all the regulators were significantly different. The degree of methylation of LGG samples was considerably lower than normal samples. These results indicate that m6A regulators could affect the epigenetic traits of patients with LGG through various mechanisms.

### Interaction Between m6A Modifications and Angiogenesis

Given that m6A modifications occur mainly in GSCs that accumulate in the periphery of neovascularization, we analyzed ARGs and m6A modifications to verify their correlation. We first conducted K-M analysis of ARGs and found that 24 out of 36 ARGs were significantly associated with survival, representative images were shown in **Figure S2**. This result confirms the key effect of ARGs in LGG and argues for the importance of vascular targeting therapy for LGG treatment breakthrough.

**Figure 2:**
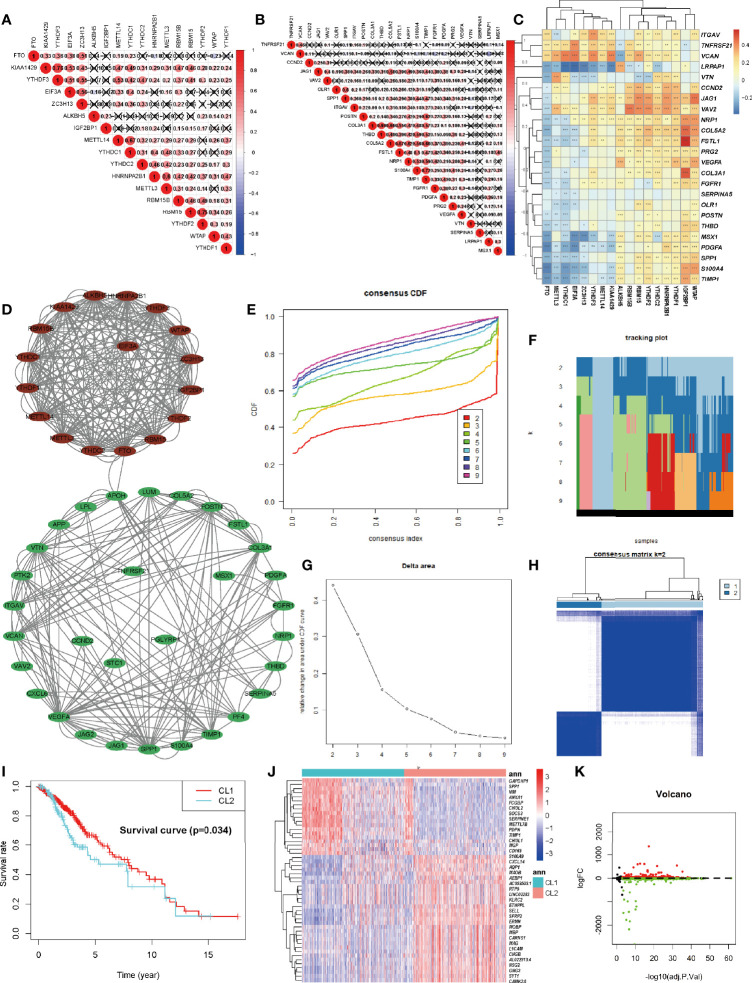
Identification and Functional Enrichment of Two Clusters of LGG. **(A)** The m6A modification-related interactions among the studied m6A regulators. **(B)** The angiogenesis-related interactions among the studied angiogenesis-related genes (ARGs). **(C)** The correlations between each m6A regulators and the expression of each ARGs using Spearman analyses. A negative correlation was marked with blue and positive correlation with red. **(D)** The protein-protein interaction between ARGs and m6A regulators (MAGs). The denseness of connection lines represented the connection strength of each node. ARGs were labeled as green dots in the circle and m6A regulators were labeled as red dots in the circle. **(E)** Consensus clustering cumulative distribution function (CDF) for k = 2-10 in TCGA-LGG cohort. **(F)** Relative change in tracking plot under the CDF curve for k = 2-10 in the TCGA-LGG cohort. **(G)** Relative change in area under the CDF curve for k = 2-10 in the TCGA-LGG cohort. **(H)** Consensus clustering matrix for k = 2 in the TCGA-LGG cohort. **(I)** Kaplan-Meier curves for two robust clusters in the log-rank test. **(J, K)** Heatmap **(J)** and volcano map **(K)** of differential expressed genes between dichotomous layers based on unsupervised clustering. The asterisks represented the statistical P-value ∗P <0.05; ∗∗P <0.01; ∗∗∗P <0.001.

Each of the 17 m6A regulators and 36 ARGs exhibited a significant self-positive correlation. Among them, KIAA1429 and YTHDF3 showed the largest correlation of 0.76, followed by RBM15 and YTHDF2 with a correlation of 0.75 **(** **Figures 2A, B** **)**. We demonstrate the correlation among the expression patterns of m6A regulators and ARGs using a correlation clustering heatmap. **Figure 2C** showed a significant negative correlation between FTO, the m6A “eraser”, and most ARGs. Macroscopically, the expression values of most ARGs and m6A regulators correlated significantly, but the correlation trends were inconsistent (positive or negative), suggesting a complex relationship between m6A modifications and angiogenesis. The study of such complex interactions may lead to unexpected results. We then retrieved the protein-protein interactions of 53 MAGs (17 m6A regulators and 36 ARGs) from the String database and found the association between APOH and FTO that were validated in the String database, while other associations were not yet known. HNRNPA2B1 seems to be the hub node of the “m6A readers”, followed by YTHDF2, and its interactions with YTHDC1, YTHDF1, YTHDF2, YTHDF3, EIF3A, and IGF2BP1 were supported both by high-throughput experimental data and by databases and published literature mining in the String database. As for “m6A writers”, METTL3/14, RBM15, and KIAA1429 might be considered as hub genes. VEGFA and SPP1 appear to be the hub genes of ARGs (**Figure 2D**). A deeper investigation of this unknown relationship may lead to unexpected gains.

### Identification and Functional Enrichment of Two Clusters of LGG

Considering the significant correlation between these regulators and ARGs, to obtain a robust classification, consistent unsupervised methodology applied in R was employed to obtain a robust ranking for subsequent analysis. To improve the accuracy of unsupervised clustering and specificity for LGG, considering the wide range of 53 genes among pan-cancers we initially screened 53 genes for univariate regression correlations with clinical prognosis **(** **Figure S4A** **)**. Finally, 12 genes with P less than 0.1 were utilized for unsupervised clustering. The consensus distributions for k (2 to 10) were displayed in the empirical cumulative distribution function (CDF) plots (**Figures 2E, F** **)**. Combined with the consensus matrix for analysis, K=2 was the most suitable choice (**Figures 2G, H** **)**. Unsupervised classes of the transcriptome data from the 529 samples with LGG revealed two clusters of samples (363 samples in one group labeled as CL1 and 161 samples in another group labeled as CL2). The consensus matrix illumined that the 12 MAGs could distinguish samples clearly, and each sample in a cluster possessed a high correlation (**Figure 2H**). To investigate the clinical prognosis differences between the two groups, a K-M survival analysis was performed and showed that the survival rate of the CL1 subgroup was significantly higher than that of the CL2 subgroup (P < 0.05, **Figure 2I**). This result indicated that these MAGs could classify the GBM samples at the prognostic level. We also tested the up-regulated DEGs in the CL2 subgroup (**Figure 2J, K** **)**. We found that out of 1674 DEGs, 220 genes were significantly associated with poor prognosis (P < 0.05, representative figures were shown in **Figure S3**). The P-value was listed in **Supplementary Table S2**. These findings suggest that the two subgroups divided by consensus cluster based on the 12 MAGs. expression are a robust classification. Annotation analysis of network differences for such stable stratification may reveal the molecular regulatory mechanisms inherent in LGG.

#### Potential Compounds Targeting the MAGs

**Figure 3:**
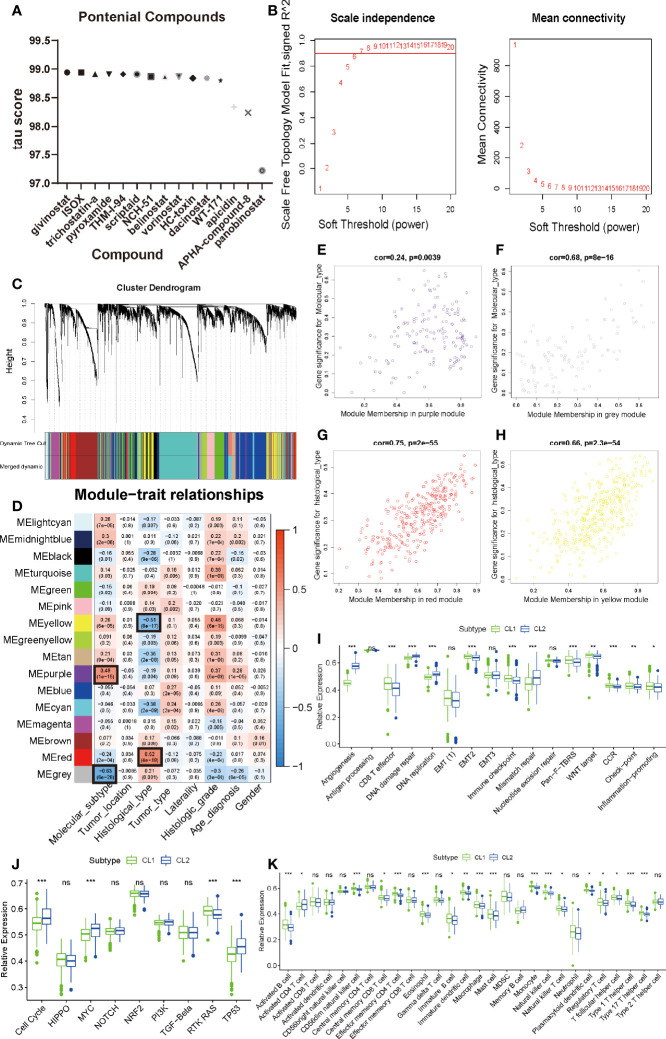
Identification of meaningful modules and key genes. **(A)** Histogram of fourteen compounds shared the CMap mode-of-action of HDAC inhibitor, sorted by descending tau score of compounds. tau, connectivity score. **(B)** Determination of soft-thresholding power in weighted gene co-expression network analysis (WGCNA). **(C)** Hierarchical clustering dendrograms of identified co-expressed genes in modules in LGG. The branches of the cluster dendrogram correspond to the 10 different gene modules. Each leaf on the cluster dendrogram corresponds to a gene Each colored row represents a color-coded module which contains a group of highly connected genes. A total of 20 dynamic modules and 16 merged modules was identified in LGG respectively. **(D)** Correlations between the gene modules and clinical traits. The correlation coefficient in each cell represented the correlation between the gene module and the clinical traits, which decreased in size from red to blue. The corresponding P-value is also annotated. **(E–H)** Scatter plot of module eigengenes in the MEpurple module with molecular subtype **(E)**, a MEgrey module with molecular subtype **(F)**, a MEred module with histological type **(G)**, a MEyellow module with histological type **(H)**. Cor was the coefficient indices and p was Pearson’s correlation. **(I–K)** The enrichment differences of typical biological processes **(I)**, oncogenic pathways **(J)** and immune cell infiltration abundance **(K)** between CL1 and CL2. The upper and lower ends of the boxes represented an interquartile range of values. The lines in the boxes represented the median value, and the dots showed outliers. The asterisks represented the statistical P-value (∗P <0.05; ∗∗P <0.01; ∗∗∗P <0.001, ns, no significant).

Considering that consistent cluster analysis revealed MAG-related malignant biological functions and pathways, compounds that target these specific targets and pathways (targeting drugs) are expected to be identified to target pathways and genes associated with m6A modification. The DEGs based on the cluster grouping were submitted to the CMap database (46). CMap mode-of-action (MoA) analysis of the top 48 compounds with 33 mechanisms that can repress the expression pattern was listed in **Supplementary Table S3** and **Figure 3A**. Fourteen compounds (Givinostat, ISOX, trichostatin-a, apicidin, and panobinostat) shared the MoA of HDAC inhibitor.

By applying the pRRophetic algorithm, we performed IC50 comparisons between high and low MASig groups for common compounds (FDA approved) and calculated their P-values (47). The results showed a significant difference in MASig for compounds such as Cyclopamine and Pazopanib, which confirms the accuracy and precision of our analysis strategy (**Supplementary Table S4**). In addition, the discovery of such compounds will provide a predictive study basis for future studies.

Recently, multiple pharmacological studies have shown that compounds acting on multiple genes or molecular pathways need to be designed (48–51). In this study, we observed similar mechanisms among different compounds, which indicated that selective therapy could target the MAGs-related phenotype of LGG.

#### Identification of Meaningful Modules and Key Genes

To further discover the differences between our cluster analysis subgroups based on MAGs, weighted co-expression network analysis (WGCNA) was applied to structure gene co-expression networks and further identify biologically meaningful modules which positively corresponded to MAG. Screened DEGs with the cutoff value (|logFC| > 1 and P < 0.05), of which 6401 were down-regulated and 3339 were up-regulated, were used to create a scale-free system (**Figure 3B**). The samples with LGG were hierarchical clustered based on Euclidean distance with FPKM count. Some samples were removed as outliers (**Figure S5A**). The patients with common clinical-pathological parameters were then added below the sample dendrogram. The sample dendrogram showed no obvious abnormal values, and the heatmap of the basic clinicopathological parameters of the samples did not include atypical patients (data was not shown). The scaleFreePlot shows the selection process of the most suitable parameter β for converting the adjacency matrix into a scale-free topology (**Figure 3B**). The threshold parameter (β = 7, R2 = 0.911) was identified by the scale-free topology criterion. 20 modules were identified by the dynamic branch cutting algorithm with minClusterSize = 30. Sixteen modules (Merged dynamic) were obtained by combining the modules whose correlation degree was greater than 0.75 (**Figure 3C**).

To analyze the connection of merged modules and clinical parameters, module Eigengenes (MEs) which can be regarded as a representative of the gene expression patterns in a module was summarized and used to calculate the correlation with clinical traits, such as molecular subtypes, tumor location, histological type, tumor type, laterality, histological grade, and gender. The heatmap revealed that two key modules (MEpurple and MEgrey in molecular subtype, MEyellow and MEred in histological type) most correlated with molecular subtype and histological type in LGG, respectively (**Figure 3D**). The MEpurple were significantly positively correlated with molecular subtype (cor = 0.49 P = 1e-15), while MEgrey were significantly negatively correlated with molecular subtype (cor = -0.63 P = 6e-28). The MEred were significantly positively correlated with histological type (cor = 0.52 P = 4e-18), while MEyellow were significantly negatively correlated with histological type (cor = -0.51 P = 8e-17). These results allowed us to select the modules of interest for further analysis.

#### Functional Enrichment Analysis of Critical Modules

**Figure 4:**
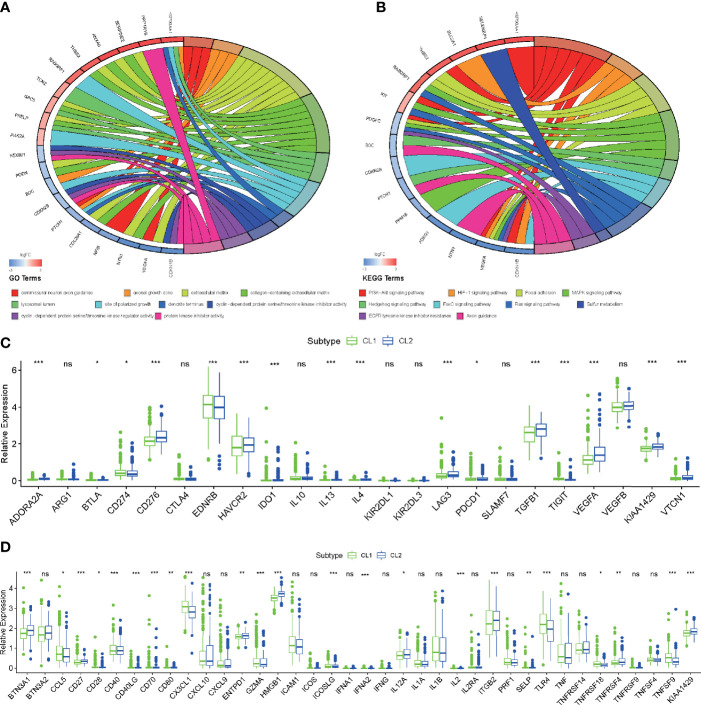
Functional enrichment analysis of key genes and critical modules. **(A, B)** Functional annotation of the hub genes in the molecular subtype modules with GO analysis **(A)** and KEGG pathway analysis **(B)**. **(C, D)** Differences in the expression of immunosuppressive **(C)** and stimulator genes **(D)** in the CL1 and CL2 subgroups. The upper and lower ends of the boxes represented an interquartile range of values. The lines in the boxes represented the median value, and the dots showed outliers. The asterisks represented the statistical p-value (*P < 0.05; **P < 0.01; ***P < 0.001, ns, no significant).

For more accurate analysis, MEs with criterion MM > 0.5 and GS > 0.5 were designated as hub genes. The functional analysis was applied in two modules (MEpurple and MEgrey) in molecular subtype and two modules (MEred and MEyellow) in histological type to explore the potential biological processes and pathways which were related to hub genes (**Figures 3E–H**). As shown in **Figure 4A**, the GO terms suggested that in the molecular subtype, the hub genes were mainly enriched in cyclin-dependent protein serine, threonine kinase regulator activity, and protein kinase inhibitor activity. KEGG terms mainly include the PI3K−Akt signaling pathway, HIF−1 signaling pathway, and MAPK signaling pathway (**Figure 4B**). The GO terms axonogenesis, axon development, and neurotransmitter secretion were enriched in MEred and MEyellow modules in histological type (**Figure S4B**). Cell adhesion molecules, Wnt signaling pathway, and Synaptic vesicle cycle as KEGG terms were enriched in MEred and MEyellow modules in histological type (**Figure S4C**). These functional annotation results for critical modules reveal a molecular phenotypic regulatory mechanism for both m6A modification and angiogenesis.

Since functional annotation analysis identified such biological processes, we speculate whether there are differences in molecular mechanisms between these two classifications that affect the prognosis of patients in both groups. We applied ssGSEA analysis to identify differences in common signaling pathways between the two groups. We found that angiogenesis, DNA replication, DNA damage repair, and DNA mismatch repair were all significantly elevated in CL2. However, in CL1, CD8 effector T cells, Immune checkpoint, Pan-F-TBRS, WNT, and Inflammation-promoting pathways all exhibited significant-high enrichment (**Figure 3I**). Previous studies have found that both m6A and angiogenesis can contribute to the immunosuppressive phenotype of TIME (39, 52–54). Here our results were consistent with this fact and laterally confirm the accuracy of our findings. Oncogenic pathways (cell cycle, MYC, and TP53) were highly enriched in CL2 also, to some extent, confirming the reason for the poor prognosis of CL2 (**Figure 3J**). To reveal the effect of both on immunity, we then analyzed immune infiltration abundance and immune-related genes. We found that the vast majority of immune cells in TIME showed high abundance in the CL1 subpopulation, which explains the high infiltration of CD8 effector cells and the better clinical prognosis in the CL1 subgroup (**Figure 3K**). This was later confirmed by the results of the analysis of immune-related genes (**Figures 4C, D** **)**. The results of these meticulous analyses of immune infiltration will undoubtedly enhance our understanding of the mechanisms of epigenetic modifications and angiogenic effects on immunosuppression. However, such a stratification strategy was only appropriate for our understanding of the intrinsic molecular mechanisms and does not apply to clinical management and practice.

#### Prognostic Value of MAGs in LGG

**Figure 5:**
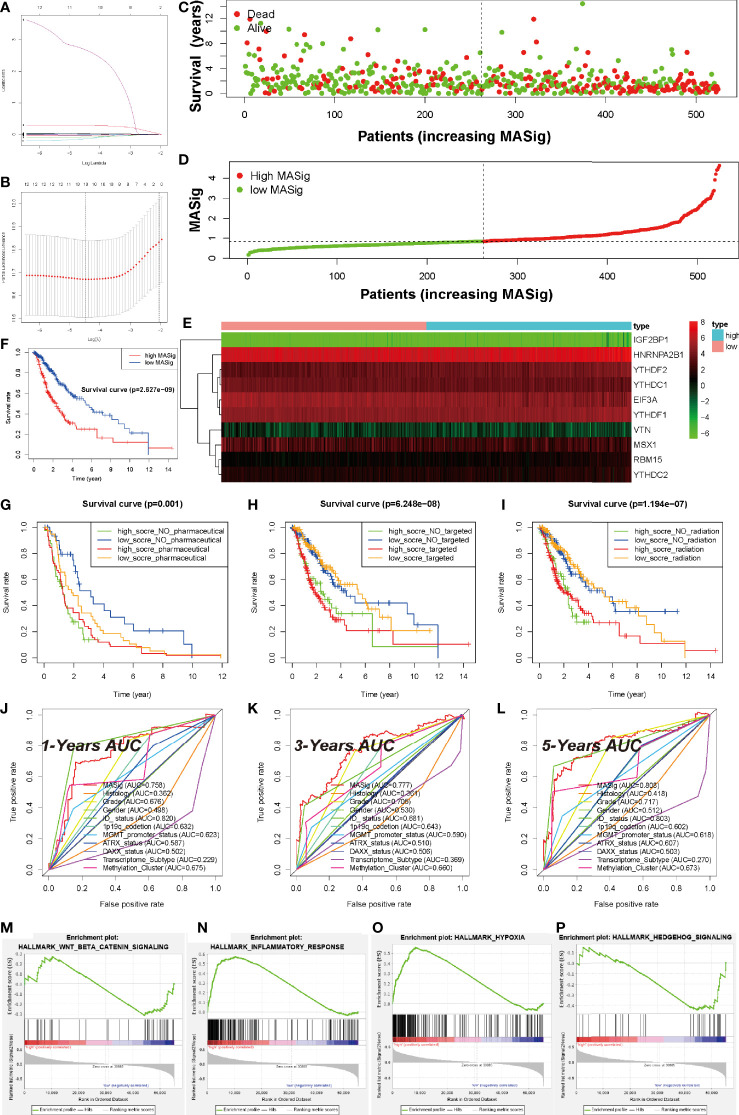
Prognostic Value of MAGs in LGG. **(A)** Ten-time cross-validation for tuning parameter selection in the TCGA-LGG cohort. **(B)** Regression coefficient profiles of identified m6A regulators in the TCGA-LGG cohort. **(C)** The distribution of survival status of MASig. **(D)** The distribution of MASig (high and low) of patients with LGG. **(E)** The correlation and distribution of hub MAGs expression values and MASig. **(F)** Survival analyses for low- and high- MASig groups in TCGA-LGG cohort using Kaplan–Meier in Log-rank test. **(G–I)** Survival analyses for subgroup patients stratified by both MASig and treatment with pharmacological chemotherapy **(G)**, targeted therapy **(H)**, and radiotherapy **(I)** using Kaplan– Meier curves in the Log-rank test. **(J–L)** ROC curves with calculated area under the curve (AUC) for risk prediction in 1 **(J)**, 3 **(K)**, 5 **(L)** years in the TCGA-LGG cohort. **(M–P)** GSEA revealed that genes with higher expression in the CL2 subgroup were enriched for hallmarks of malignant tumors.

To measure the prognostic value of the MAGs, univariate Cox regression and LASSO regression were applied to the FPKM expression pattern. Based on the information displayed in **Figure S4A**, 12 of 53 MAGs exhibited a correlation with the OS. To construct the prognosis gene-signature, these 12 genes were used as candidate genes for lasso regression analysis based on the least square method. In the cross-validation process (**Figures 5A, B** **)**, lambda. Min = 1.16544352 was considered as the optimal value, log (lambda) = -4.5 (Partial Likelihood Deviance was minimum). The regression coefficient hence was calculated and displayed in **Figure 5A**. These results show that all of the 10 candidate genes were the optimal genes for constructing the gene-signature (except for FTO and ALKBH5).

To test the performance of the prognostic gene-signature, we calculated the risk score (labeled as MASig) of each sample and conducted the further analysis. The MASig of samples were divided into two levels based on the median value. The survival status, OS, and risk score levels of patients were shown in **Figures 5C, D**. Also, the expression patterns of the six optimal genes were divided into two categories by cluster analysis based on Euclidean distance and were negatively correlated with risk grouping (**Figure 5E**). These results indicate that survival rates were lower in the high-MASig group. Then, we conducted a K-M survival analysis of the two risk groups and found that there was a significant difference in survival rates between the two risk groups **(** **Figure 5F**).

To further explore the reflectivity of MASig on the prevailing therapeutic paradigm of LGG nowadays, we did a more in-depth analysis of them. The results showed that whether patients in the High-MASig group received drugs, radiotherapy, or targeted therapy, their survival time was inferior to that of the Low-MASig group (whether or not the Low-MASig group received treatment, **Figures 5G–I**). This also reflects the difference in the sensitivity of MASig to the treatment modality. Since MASig can significantly distinguish LGG patients’ sensitivity to treatment, can they be used to predict survival duration? Notably, the ROC curve and AUC were performed and calculated, respectively. The 1-year AUC was 0.758 for MASig. The 3-year AUC and 5-year AUC for MASig were maximums of 0.777 and 0.808, respectively (**Figures 5J–L**). Together, these findings indicate that the gene-signature shows excellent accuracy for prognosis prediction.

Furthermore, GSEA revealed that the malignant hallmarks of LGG, including inflammatory response (NES=1.81, normalized P-value < 0.001, q-value= 0.038), Wnt-beat-catenin signaling (NES=1.70, normalized P-value < 0.001, q-value= 0.051), hypoxia (NES=1.96, normalized P-value < 0.001, q-value= 0.022), and Hedgehog (NES=1.96, normalized P-value < 0.001, q-value= 0.021), were significantly enriched in the high-MASig subgroup (**Figures 5M–P**). All these results revealed the malignant related processes and pathways of MAGs in LGG.

#### Comparisons of Somatic Mutations Under Different MASig

**Figure 6:**
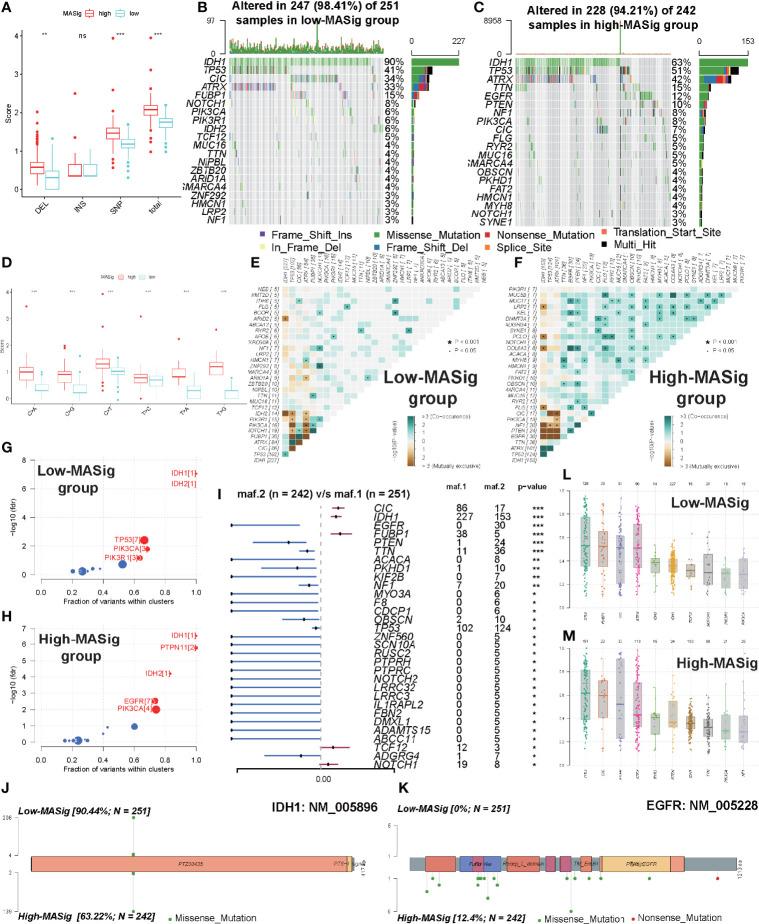
Comparisons of somatic mutations under different MASig. **(A)** The differences in the fraction of deletion (DEL), insertion (INS), single-nucleotide polymorphism (SNP), and total variance between high and low MASig subgroups. **(B, C)** Waterfall plots about the mutation distribution of the top 20 most frequently mutated genes between low **(B)** and high **(C)** MASig subgroups. **(D)** Differences in the fraction of six types of single-nucleotide variants (SNV) between high and low MASig subgroups. **(E, F)** The correlation heatmaps about correlations of 30 top mutated genes between low **(E)** and high **(F)** MASig subgroups. The color and symbol in each square represented the statistical significance of the exclusive or co-occurrence for each pair of genes. **(G, H)** Driver genes of the low **(G)** and high **(H)** MASig subgroups. The horizontal line of the box plot represented the median values. **(I)** The forest plot about the differential mutation profiles of 30 most frequently mutated genes between high and low MASig subgroups. **(J, K)** The lollipop plots displayed the differential distribution of mutation loci and types for IDH1 **(J)** and EGFR **(K)**. In the box plots and the forest plot, p<0.05 was indicated by “*”, p<0.01 was indicated by “**”, p<0.001 was indicated by “***”, and no significant was indicated by ns.

To further reveal the genetic mechanisms behind how MAGs impacted the malignant characteristics and oncogenesis, we investigated the differences between the high and low MASig subgroups at the genomic layer. From the global perspective, the high MASig subgroup had a higher incidence of DEL, SNP, and total variants (**Figure 6A**). Also, among all six types of SNV, the mutation rate was significantly higher in the high MASig subgroup (**Figure 6D**). Next, we counted the forms of mutations and found that the missense mutation was predominant in the LGG samples (**Figure 6B**). These results suggested that the process of genomic variation might have some regularities during the formation processes of m6A modifications and angiogenic. Additionally, elevated SNP and DEL levels were correlated with cancer susceptibility and progression (55–57). In addition, VAF levels of genes differed between high and low MASig subgroups (**Figures 6L, M** **)**. Altered VAF levels of some genes, such as TP53, were thought to be associated with the prognosis and outcome of oncology treatment (58). Therefore, it was necessary for us to explore the regularity of genetic heterogeneity between the high and low MASig subgroups and reveal their potential biological meanings.

Firstly, we investigated the differences in the mutation profiles between the two MASig subgroups. We found three of the 30 most frequently mutated genes had a higher mutation rate in the low MASig subgroup (**Figures 6I** and **S6A**). Besides the mutation rate, the mutated loci also differed between high and low MASig subgroups. Among the four genes with the most significant differences in mutation rate, mutated loci and types in EGFR, CIC, and FUBP1 were diverse except for IDH1 (**Figures 6J, K**, and **S6A–C**). In addition, by comparing the mutation profiles, we found that the mutation rate of IDH1, EGFR, CIC, and FUBP1 were all more than 10% in both high and low MASig subgroups (**Figures 6B, C** **)**. Also, IDH1 and epidermal growth factor receptor (EGFR) were identified as driver genes in the high MASig subgroup. Importantly, IDH1 was a cancer driver gene in both groups. (**Figures 6G, H** **)**. Oligodendrogliomas were characterized by a common 1p/19q deletion and mutations in CIC, FUBP1, Notch1, and TERT promoters. Our findings on CIC and FUBP1 mutations may be able to confirm this view. This finding may be attributed to the satellite phenomenon of oligodendroglioma (tumor cells accumulate around blood vessels and interact closely with newly generated vessels) (59). Further analysis showed that PIK3CA was similarly identified as driver genes in both groups (**Figures 6G, H** **)**. Therefore, the functions performed by IDH1 and PIK3CA in oncogenesis and progression of LGG were relatively stable at different mutation levels. To better understand the mechanisms and underlying regularities in the establishment of genetic heterogeneity, we analyzed the correlations between the top 30 mutated genes. Interestingly, in the high MASig subgroup, most of the genes exhibited the co-occurrence landscape. However, seldom do genes interact with each other in the low MASig subgroup (**Figures 6E, F** **)**.

Finally, we analyzed the enrichment level of oncogenic signaling pathways between the high and low MASig subgroups. Samples with high MASig were significantly highly enriched in all of these pathways (**Figures S6D, E** **)**. This finding revealed some of the genetic mechanisms about how m6A modifications and angiogenesis affected the malignant characteristics of LGG. For example, activation of RTK-RAS, TP53, WNT, and PI3K signaling pathways were strongly correlated with oncogenesis and progression of cancers, contributing to a higher grade and worse prognosis (60–63). TGF-Beta, NOTCH, and WNT pathways were involved in regulating the EMT process (64, 65). In addition, Hippo and WNT pathways were associated with stemness generation, and the NOTCH pathway was involved in promoting the formation of the immunosuppressive microenvironment (66–68). In summary, because of genetic heterogeneity, oncogenic signaling pathways were differentially enriched between samples with different subgroups, which further revealed the genetic mechanisms potentially involved in how m6A modifications and angiogenesis affected the malignant characteristics and oncogenesis of LGG.

#### The Role of the MASig in Anti-PD-1/L1 Immunotherapy

Entering, strategies to block immune checkpoints, such as PD-1 and PD-L1, have been shown to be promising therapeutic approaches that allow patients to achieve therapeutic survival benefits. Considering that some patients are resistant to immunotherapy, the identification of immunotherapy-sensitive biomarkers has become a focus of contemporary research (41, 50–53, 69). Based on our findings of significant correlation between MASig and immune components, we tested the predictive and investigative capacity of MASig for immunotherapy. After analyzing the expression pattern of MAG in the (IMvigor210) cohort we found that the PD-L1 blockade with atezolizumab group and the control group showed significant differences (**Figures S7A, B** **)**. Among them, high MASig possessed a worse prognosis (**Figure S7C**). In addition, MASig also had a predictive ability for survival (**Figure S7D**). Most human solid tumors exhibit one of three distinct immunological phenotypes: immune inflamed, immune excluded, or immune desert (70). We found that the desert phenotype had the highest MASig and was significantly different from the other two phenotypes (**Figure S7E**). It is known that inflamed cancers are most sensitive to immunotherapy (71). We also found that inflamed cancers had the highest MASig, which echoes the above fact (**Figure S7E**). These findings implies that immunotherapy cannot modulate the expression pattern of these MAGs, therefore, we hypothesized that the MASig can reflect the sensitivity of immunotherapy. Next, we analyzed the differences in MASig in samples from patients with various responses to immunotherapy and found that the low-MASig group mainly contained samples from patients in the response group (**Figure S7F**). Tumor neoantigen burden, as a molecular marker that more directly reflects the response to immunotherapy, was introduced to explore the survival benefits between patients with various MASigs. We found that the MASig was significantly negatively correlated with the number of mutations (TMB, **Figure S7G**). Moreover, we determined the enrichment of canonical oncogenic pathways and found that most of the pathways were highly enriched in the high-MASig group (**Figures S7H, I** **)**. The above findings suggest that the MASig not only can reflect the sensitivity of patients to immunotherapy but also is related to the progression of cancer.

## Discussion

m6A modification, as a hot spot in the epigenetic field nowadays, has been systematically analyzed in numerous studies. However, the interaction between m6A modification and angiogenesis is still sporadically reported (72–74). The systematic analysis of both remains a macroscopic landmark. In this study, we demonstrated the potential malignant mechanism of the common m6A regulator and ARGs in the development of LGG and examined their predictive value for the prognosis of LGG. We first identified significant correlations between these 17 regulators and ARGs and discussed the internal interactions and correlations of these MAGs. Under this premise, we then identify and analyze two robust subgroups by consensus clustering and find out their potential impact on patients with LGG. Also, WGCNA was implemented to identify the modules related to the clinical characteristics of interest and to select these hub genes in the modules. After the functional annotation of these modules, we found the potential mechanisms related to the pathological types and molecular subtypes. Then, we searched the Cmap database to identify the compounds specifically targeting m6A and angiogenic phenotype. We build a gene-signature prediction model with high quality and accuracy based on 12 MAGs and corresponding MASig. Finally, based on MASig, we characterized their somatic mutant subtypes to delve into their underlying mechanisms. We found the correlation between MAGs and clinical characters, which indicated that by analyzing the expression patterns of MAGs, we could find the value of prognosis, diagnosis, and treatment. Our specific and comprehensive analysis of the value of MAGs in LGG can provide guidance for the future study of LGG and facilitate the clinical work of LGG.

Angiogenesis was critical to tumor development and progression and was essential for tumor cell proliferation. VEGF plays an important role in this process. Overexpression of EGFR and EGFRvIII was frequently observed in LGG and was a key factor in LGG progression. LGG was the most common malignant brain tumor in adult CNS and remains incurable with dismal median survival. Targeting EGFR for LGG is an attractive therapeutic strategy. For example, Cabozantinib exerts its antitumor effects by targeting VEGFR2 and inhibiting angiogenesis n hepatocellular carcinoma (75). Regorafenib also inhibits tumor angiogenesis by acting on VEGF and has shown significant survival benefits as second-line therapy in hepatocellular carcinoma (76). Although the EGFR inhibitor gefitinib was effective in the treatment of non-small cell lung cancer, it was not efficacious in GBM (77). The PDGFR inhibitor imatinib also has extremely limited efficacy in recurrent and newly diagnosed GBM (78). Everolimus, an mTOR inhibitor, was not significantly effective in both recurrent and newly diagnosed GBM (79). Tipifarnib regulated transcription and modification of the Ras gene and affects cell proliferation and apoptosis but has no significant efficacy in newly diagnosed GBM (80). Apparently, resistance to EGFR inhibitors in LGG patients has stimulated the development of multi-targeted or epigenetic agents or the use of combination drugs for the treatment of gliomas. Epigenetic modifications were also closely associated with proliferation, invasion, metastasis, and prognosis of glioblastoma (41, 52, 53).

The decisive role of epigenetics in the traditional sense, including only DNA and histone inheritance, in tumor progression and treatment, has been confirmed (81). 163 RNA modifications retrieved from MODOMICS (2017 update), first discovered in 1970, have been identified in almost all forms of natural cellular RNA (9, 82). m6A is mainly concentrated near the stop codon and 3’untranslated terminal region (UTR) and is translated at 5’UTR. These findings reveal that the m6A regulator regulates the synthesis, metabolism, transcription, and translation of mammalian RNA (83, 84). In colon cancer cells, the m6A reader IGF2BP3 promotes neovascularization by recognizing and binding to the m6A modification site in VEGF mRNA and promoting its expression (85). METTL3 promotes the maturation of miR-143-3p to target the vasopressor (VASH) 1 promoter and inhibit its expression in lung cancer (43). VASH1 mediates miR-143-3p-induced angiogenesis by destabilizing VEGFA proteins (86). IGF2BP3 directly recognizes and binds to the m6A modification site of METTL3-mediated HDGF mRNA and enhances the stability of HDGF, thereby promoting gastric tumor angiogenesis (87). In addition, YTHDF2 inhibits the normalization of tumor vasculature in hepatocellular carcinoma cells by increasing the attenuation of IL11 and SERPINE2 mRNAs (88). The link between m6A modification and angiogenesis was identified in our analysis. PPI validated their interaction in glioma. These findings may lead to new research directions for vascular-targeted therapy of gliomas, as m6A modifications in angiogenesis have not been addressed, at least until now.

TCGA has now emerged as an efficient and promising tool for elucidating gene-level alterations in 33 solid tumors by generating comprehensive data consisting of the epigenome, transcriptome, and proteome, as well as histopathology and various standard clinical parameters (89). The resultant resources allow us to comprehensively analyze the impact of MAGs on the clinical prognosis of LGG, the molecular mechanisms affecting prognosis, and the construction of further clinical prediction models. Based on the current convenient resources for many tumors (glioma, GBM, lung cancer, etc.), the comprehensive analysis of m6A and angiogenesis have been reported in different depths (90–93). This study describes the expression pattern of MAGs in LGG and their crucial role in LGG development by retrieving the TCGA-LGG dataset. Research of mRNA modification is an emerging field as yet, and the significance of this epigenetic marker in affecting tumorigenesis is only just beginning to be recognized. Recently, a vital study reported that ALKBH5 elevated in GBM stem cells and maintained tumor initiation through FOXM1 expression and cell proliferation (93). Although m6A is the most abundant in brain tissue, there are only sporadic reports on brain development or brain disease (mostly about GBM), while reports of LGG have not been reported so far (84). This study reveals a causal link between the mRNA m6A regulators and the occurrence of LGG, which represents an important step in the direction of the therapeutic strategies by targeting MAGs in tumors, their upstream regulatory factors, downstream targets, or related malignant pathway.

Apparent modification of RNA has become a rapidly developing research discipline in oncology. With the gradual understanding of m6A modification, it will bring great promise for the therapeutic of human diseases. The dynamic reversibility of m6A powerfully demonstrates the critical role of RNA modification (94). Such a function might be necessary during the proliferation, invasion, and malignant process of tumor cells. Indeed, substantial differences in m6A content between different immortalized cell lines, especially cancer cell lines, have been reported, but their channels for regulating cell immortality still need to be clarified (84). A recent study demonstrated that FTO/m6A/MYC/CEBPA signaling plays a key role in leukemic (28). JAK1/STAT5/C/EBP β pathways (95) and the IL-7/STAT5/SOCS pathways were also reported to be related to BDMS and T cells, respectively (96). However, in cancer research, the m6A-related pathway is still sporadic, and other potential carcinogenic pathways are being explored. In this study, through functional annotation of the results of consistent clustering, a multitude of possible pathways and biological processes in LGG were discovered, including RNA splicing, mRNA processing, spliceosome, MYC target, and Wnt-beat-catenin signaling. These analytical results provide predictive evidence for subsequent experimental verification, although experiments on the interaction between m6A and LGG have not been retrieved so far.

Although consensus clustering and differential analysis provide enormously detailed information, only the application of WGCNA enables us to discriminate the correlation patterns among genes, and further acquire the modules and hub genes related to clinical phenotypes and molecular phenotypes. The prognostic model of GBM constructed by WGCNA demonstrated better predictive power than other strategies (97). Furthermore, distinct pathways have been distinguished between ADC and SCC by various WGCNA modules (98). Since there were only sporadic reports about the molecular subtypes and pathological types of m6A and angiogenesis in tumors, these findings could help us to understand the potential interaction of m6A modification on LGG clinical characteristics more comprehensively. In addition, macro-level correlations between m6A and angiogenesis have been revealed, and more detailed studies are worth being anticipated.

Conventional computational methodologies have been used to determine the prognosis of LGG patients based on gene expression (53, 99–101). When cancer pathway genes are used as input variables, expression regression modeling using lasso strategy performs better than whole-genome input (102). Compared with the previous gene-signature from long non-coding RNA, immune-related RNA, our gene-signature based on MAGs was more convincing and feasible. Importantly, the predictive power of MASig was more reliable than other common clinical traits. It was also worth noting that the GSEA analysis based on MASig revealed a potential mechanism for prognosis. As far as we know, this prognostic gene-signature has not been reported in LGG so far and may provide theoretical guidance for the development of new clinical management strategies. We developed and validated gene-signature derived from MAGs to provide personalized survival assessments for newly diagnosed LGG patients. This prediction system is useful for clinicians to advise patients and their families on treatment decisions, follow-up, and prognostic decisions.

The present study is deficient due to the absence of extensive biological validation, which needs to be correlated by numerous scholars. Herein, we reveal a synergistic effect between m6A modification and angiogenesis-targeted therapy. In addition, since m6A modifications also play an important role in mediating cancer response to chemotherapy, radiotherapy, immunotherapy, and targeted therapies, targeting m6A regulators could also be applied clinically together with chemotherapy, radiotherapy or immunotherapy to achieve better cancer treatment in the near future. Targeting dysregulated m6A regulators by effective inhibitors (or targeting mutated or dysfunctional m6A sites by targeted external transcriptome editing) alone or in combination with other therapies may have potential therapeutic potential for various types of cancers, especially those that were resistant to existing therapies.

Taken together, our results presented a comprehensive characterization of m6A modification and angiogenesis in LGG based on bioinformatics and deep learning analysis. The strengths of the approach are that it leverages features of MAGs across LGG that reflect tumor malignant signaling, biological process, and clinical phenotypes of interest, as well as targeted therapeutic agents. This study also provides strategies for the analysis of the predicting prognostic potential of MAGs based on computational methodologies. Findings based on these strategies may facilitate the development of an objective diagnostic system to quantify patient survival and other outcomes, in some cases, molecular subtypes, which can reduce sequencing costs. Finally, experimental studies of these MAGs will aid further comprehending the potential interactions between m6A modification and angiogenesis in LGG and improve clinical outcomes.

## Data Availability Statement

Publicly available datasets and online tools were utilized in this study. These resources could be found here: https://portal.gdc.cancer.gov/. https://www.proteinatlas.org/. https://clue.io/.

## Author Contributions

BL conceived and designed the study and drafted the manuscript. JD and KH provided analytical technical support. FW and NW participated in the production of charts and pictures. All authors contributed to the article and approved the submitted version.

## Funding

This work was funded by the Natural Science Foundation of China (No. 61575058) and the Basic Public Welfare Research Project of Zhejiang Province (LGF22H090027).

## Conflict of Interest

The authors declare that the research was conducted in the absence of any commercial or financial relationships that could be construed as a potential conflict of interest.

## Publisher’s Note

All claims expressed in this article are solely those of the authors and do not necessarily represent those of their affiliated organizations, or those of the publisher, the editors and the reviewers. Any product that may be evaluated in this article, or claim that may be made by its manufacturer, is not guaranteed or endorsed by the publisher.
