# Supplementary material for: Identification of Implications of Angiogenesis and m6A Modification on Immunosuppression and Therapeutic Sensitivity in Low-Grade Glioma by Network Computational Analysis of Subtypes and Signatures
Source: Front Immunol. 2022 Apr 27;13:871564. doi: 10.3389/fimmu.2022.871564 (PMC9094412; doi:10.3389/fimmu.2022.871564)
Supplement: Supplementary file 1 [file DataSheet_1.docx]

# Supplementary Figures for

# Machine-learning recognition of subtypes and signature of m6A modification and angiogenesis with potential implications in immunosuppression and therapeutic sensitivity of low-grade glioma

**Figure S1. Degree of methylation and CNV of m6A methylation regulators and clinical parameters**


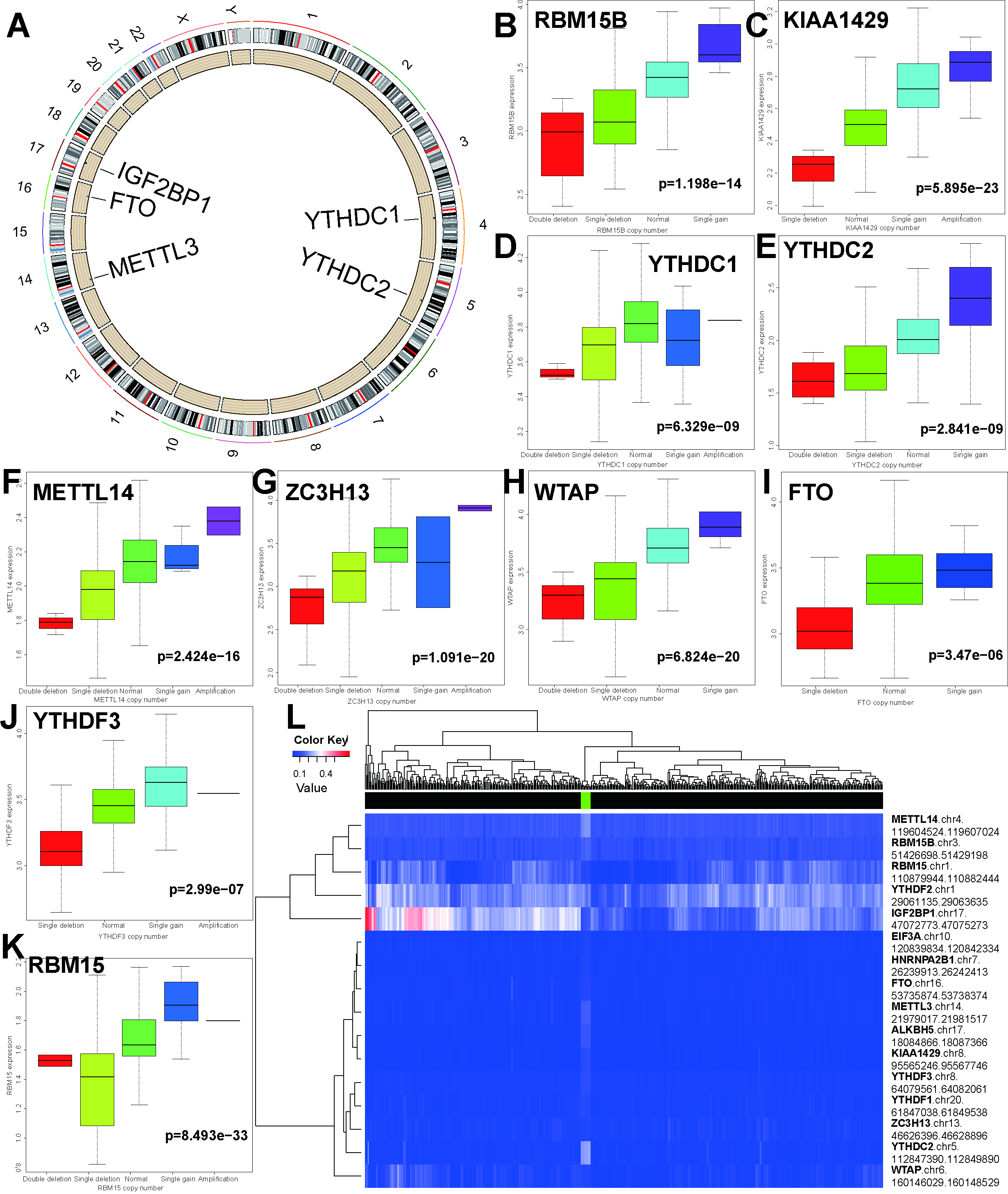


**(A)** Circle plot of differential CNV of m6A methylation regulators. The black dot in the outer ring indicates amplification, while the red dot in the inner ring indicates deletion. **(B-K)** Functional plotting of the corresponding mRNA level in relation to genetic status of RBM15 **(B)**, KIAA1429 **(C)**, YTHDC1 **(D)**, YTHDC2 **(E)**, METTL14 **(F)**, ZC3H13 **(G)**, WTAP **(H)**, FTO **(I)**, YTHDF3 **(J)**, and RBM15 **(K)** of LGGs. **(L)** The result heatmap contains methylation data of 17 transcripts from 544 samples of 450k. In the heatmap, rows represent transcripts and columns represent samples (green color represents normal profiles, black represents disease profiles).

**Figure S2. Representative images of ARGs which were significantly associated with survival.**


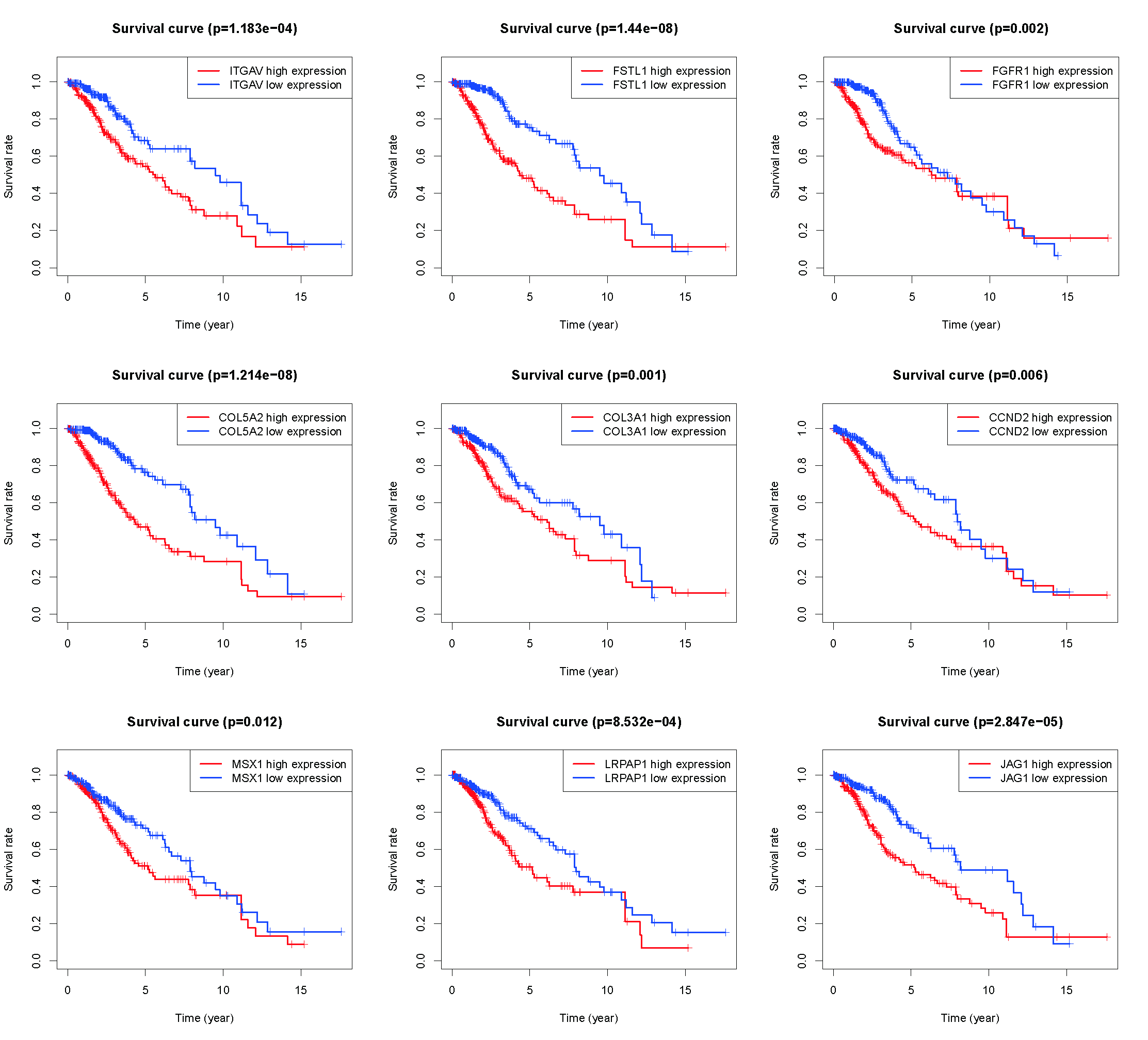


**Figure S3. Correlation of expression of individual DEGs of OS.**


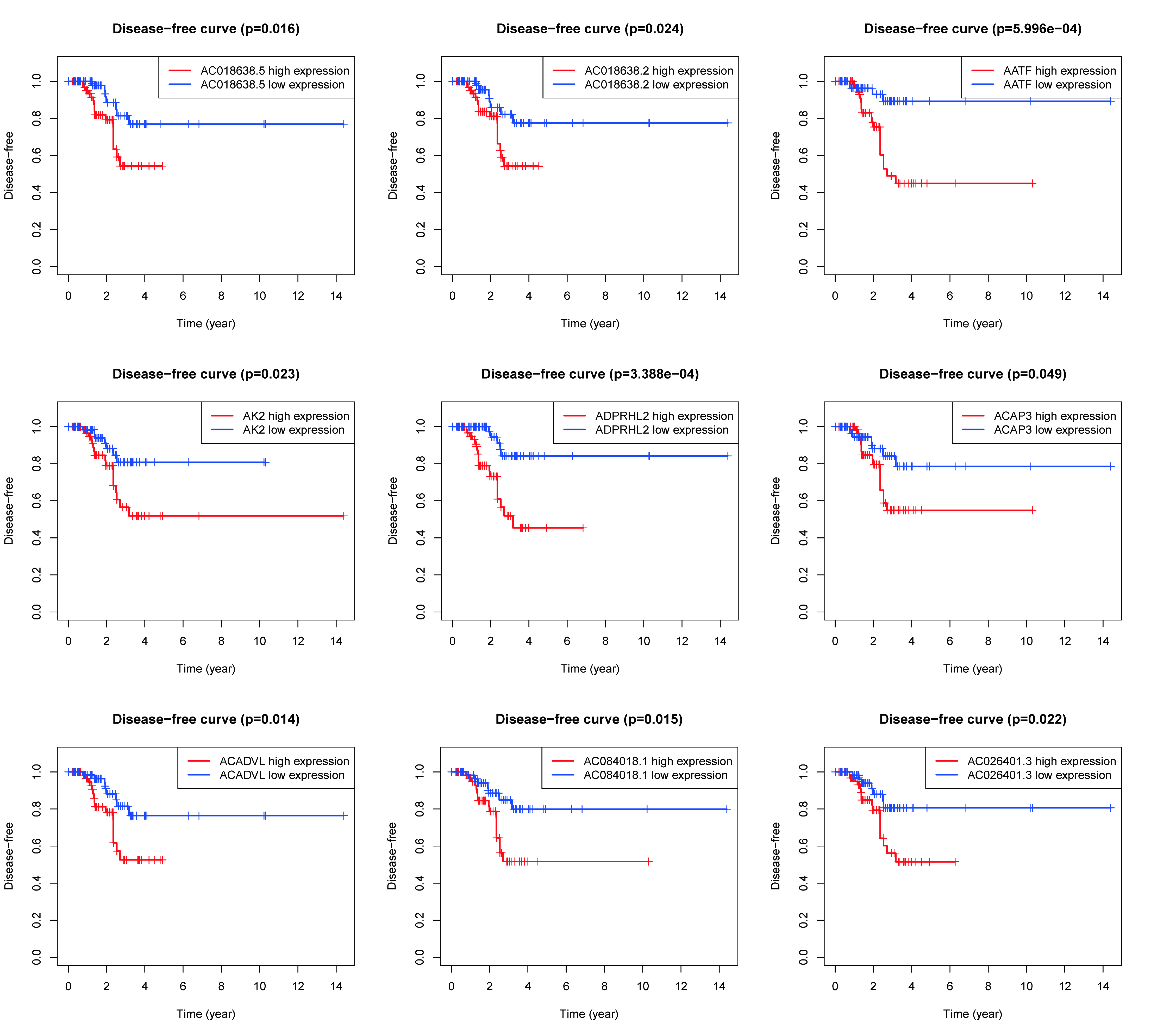


Kaplan-Meier curves were generated for represented DEGs extracted from the comparison of groups of high (red line) and low (blue line) gene expression.

**Figure S4. Functional annotation of the hub genes.**


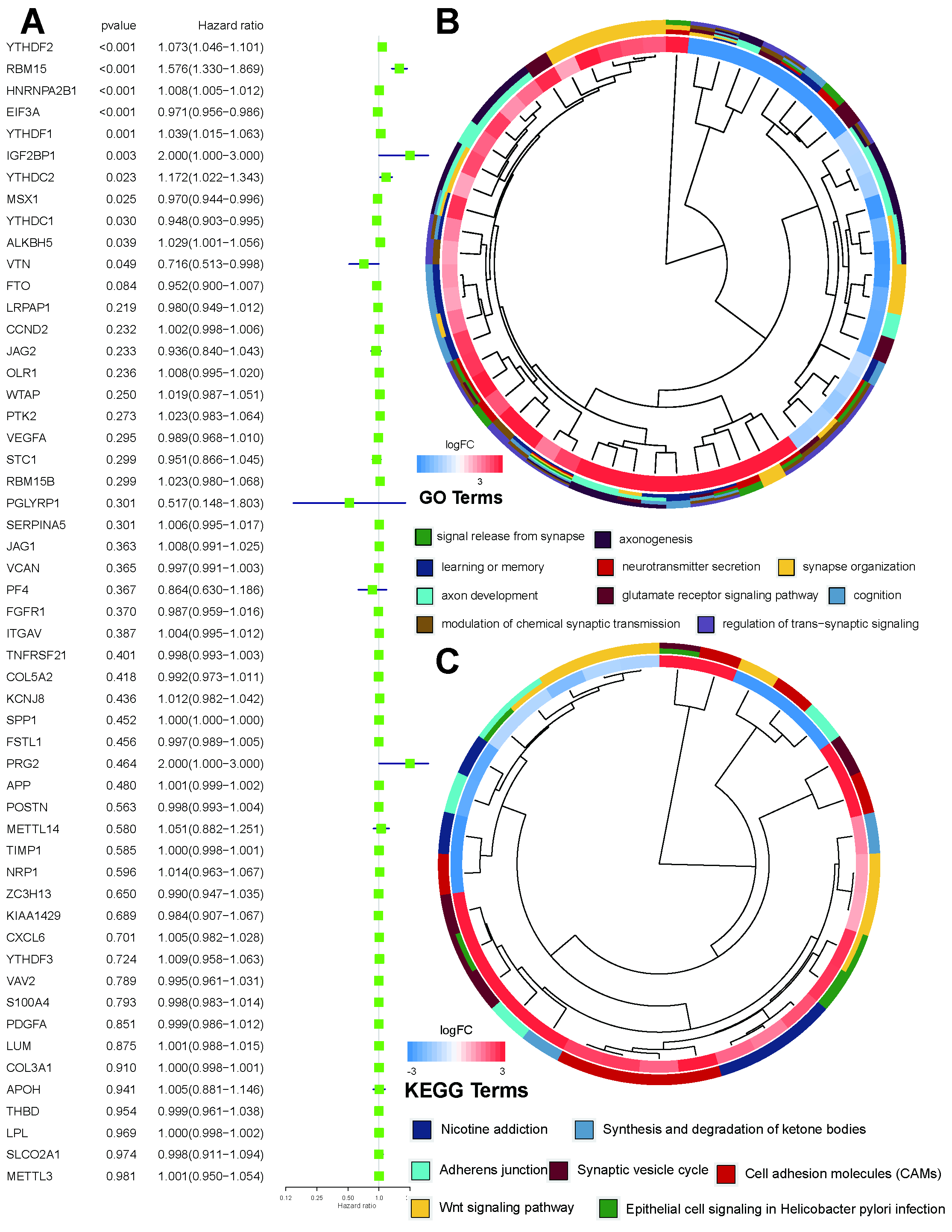


(**A**) The hazard ratios (HR), 95% confidence intervals (CI) of MAGs calculated by univariate Cox regression (**B, C**) Functional annotation of the hub genes in the molecular modules with GO analysis (**B**) and KEGG pathway analysis(**C**).

**Figure S5. Clustering of samples and removal of outliers.**


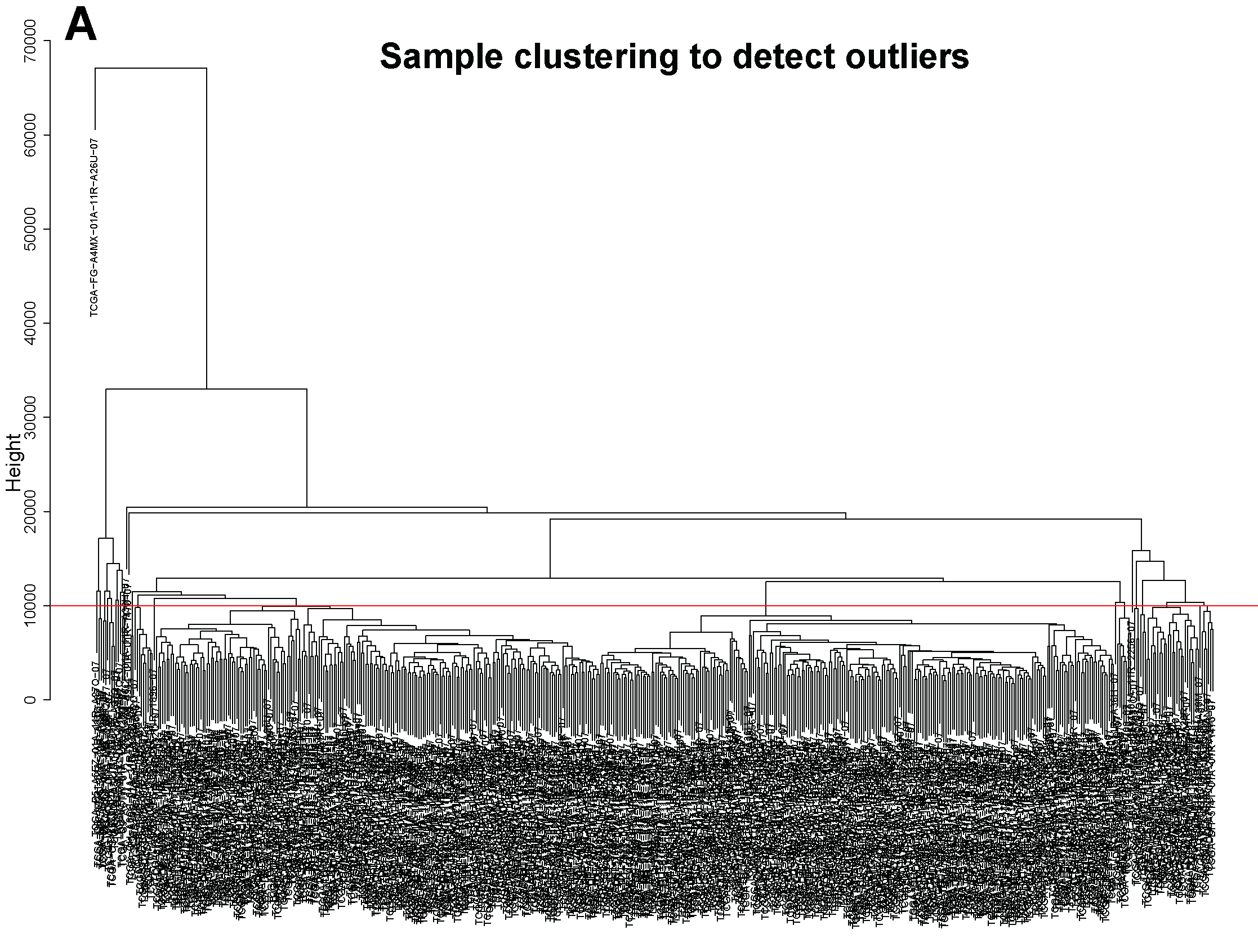


**Figure S6. Gene-signature performance measurement**


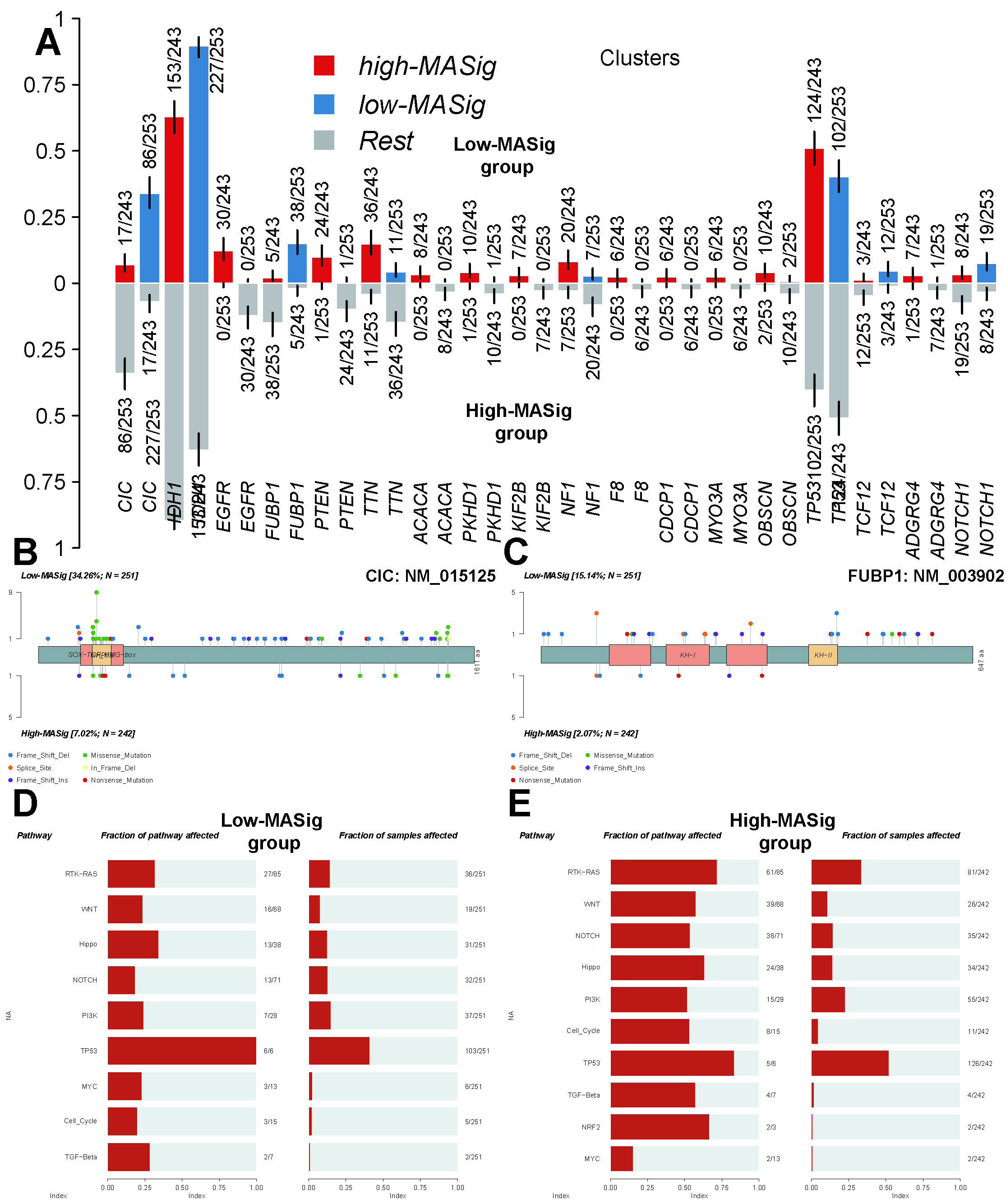


(**A**) Characteristic mutant genes of different MASig subgroups. Red represents the High group and green represents the Low group. **(B, C)** The lollipop plots displayed the differential distribution of mutation loci and types for IDH1 **(B)** and EGFR **(C)**. (**D, E**) The enrichment levels of oncogenic signaling pathways in high **(D)** and low **(E)** MASig subgroups.

**Figure S7. The role of the MASig in anti-PD-1/L1 immunotherapy**


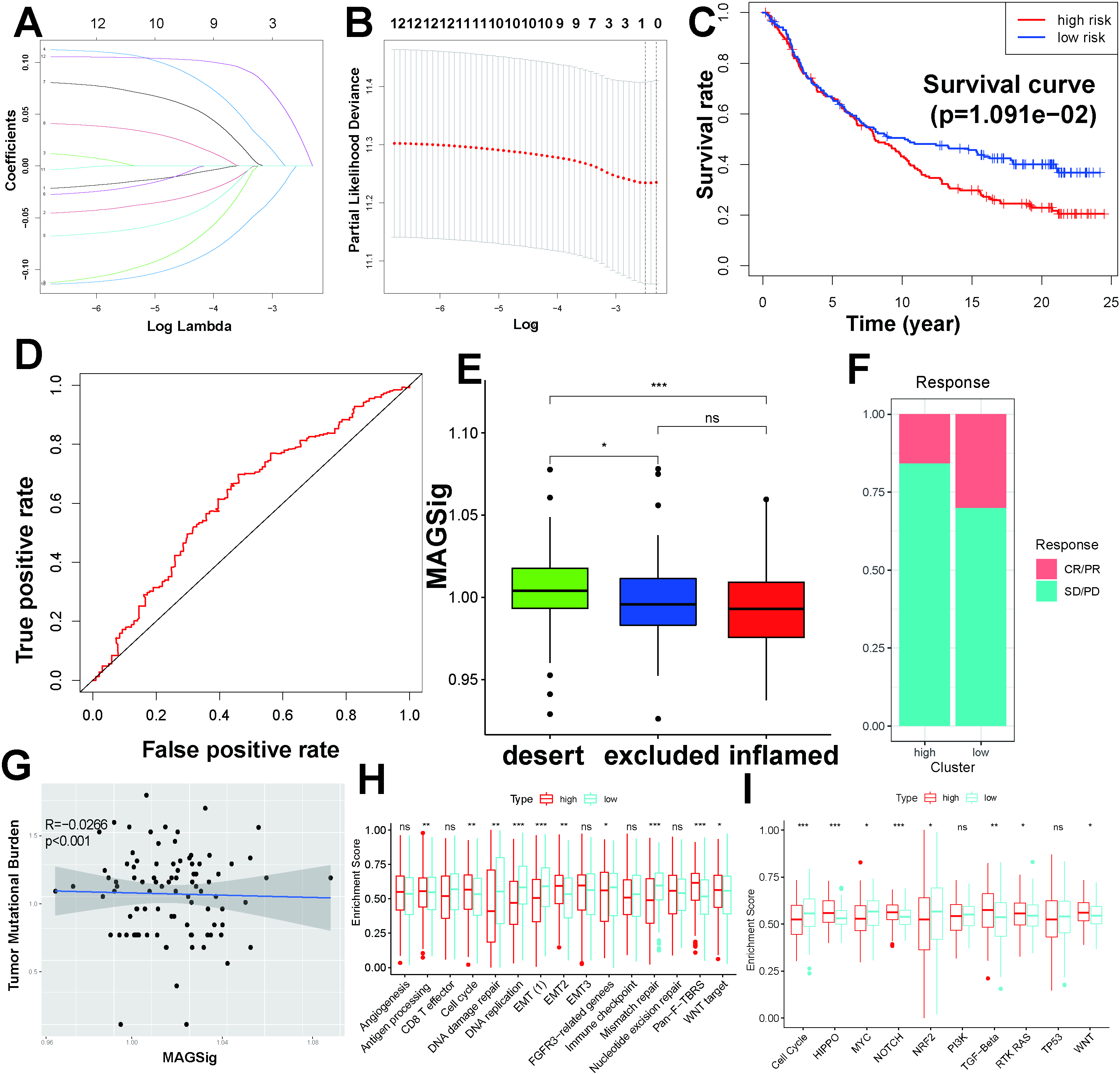


(**A**) Ten-time cross-validation for tuning parameter selection in the IMvigor210CoreBiologies cohort. (**B**) Regression coefficient profiles of identified m6A regulators in the IMvigor210CoreBiologies cohort. (**C**) Survival analysis of low- and high-m6AScore group with LGG samples in the IMvigor210CoreBiologies cohort in the Log-rank test. (**D**) ROC curves with calculated AUCs for risk prediction in 20 year. (**E**) The proportion of patients in the IMvigor210CoreBiologies cohort with clinical response in the low- or high-MASig groups. (**G**) The negative correlation between the MASig and tumor mutational burden. (**H, I**) Differences in typical biological processes (**H**) and 10 oncogenic pathways (**I**) with 335 genes between low- and high-m6AScore groups in the IMvigor210CoreBiologies cohort.
