# Supplementary material for: Identification of Implications of Angiogenesis and m6A Modification on Immunosuppression and Therapeutic Sensitivity in Low-Grade Glioma by Network Computational Analysis of Subtypes and Signatures
Source: Front Immunol. 2022 Apr 27;13:871564. doi: 10.3389/fimmu.2022.871564 (PMC9094412; doi:10.3389/fimmu.2022.871564)
Supplement: Supplementary file 3 [file Table_1.docx]

## METHOD

### Data collecting

The RNA-sequencing transcriptome per kilobase million (TPM) data with masked copy number segment (529 LGG and 37 normal samples) were downloaded from the TCGA database (https://portal.gdc.cancer.gov/repository) and also included the corresponding clinical parameters, pathological types, and molecular subtypes. Owing to patients with LGG might usually relapse before death and, hence, more clinical events were taking place during the follow-up time, disease-free interval (DFI) might typically be expected better clinical endpoint choices for the subsequent research than overall survival [1]. Notably, the curated survival data with LGG patients, including DFI, disease-free status, and disease-free rate, was collected from the Pan-cancer Atlas paper [1]. The clinicopathological parameters for the TCGA samples were summarized in Supplementary Table S1. These data were updated as of January 26, 2020. The Ensemble ID in the expression matrix was converted into the Gene Symbol by the script in Perl (http://www.perl.org/). Genes with an average expression of less than 0 and samples with a DFI of less than 90 were eliminated in this study. All gene expression data were log2-transformed. The ARGs set was downloaded from MsigDB and REACTOME databases (hallmark-angiogenesis, which includes 36 genes upregulated during the formation of tumorigenic blood vessels) [2, 3]. All operations followed the approval of the Ethics Committee of Shandong First Medical University (NCFC: NO.2022-887)

### Mutation and interactions of m6A regulators

The online cBioPortal (https://www.cbioportal.org/) tool was employed to retrieve the mutation pattern of the 17 genes in the LGG cohort. Immunohistochemistry (IHC) staining of m6A regulators in LGG was obtained from Human Protein Atlas (HPA) [4]. Protein-protein interactions (PPI) among MAGs were illustrated using STRING [5] and reprocessing via Cytoscape [6].

### Evaluation of the associations between the expression of regulators and clinical outcomes, CNV, and the degree of methylation in LGG

17 widely reported and accepted m6A regulators were identified from various studies [7, 8]. The adjusted P-value for multiple testing was applied by the Benjamini–Hochberg (BH) method to explore the association between the designated m6A regulators and age (≥60 or <60), sample type (Normal or Tumor), tumor type (Primary Tumor and Recurrent Tumor), WHO grade (G2 or G3), KPS score (≥80 or <80), and gender (Female or Male). The One-way ANOVA test was used to detect the significance between m6A regulators and histological type (Oligodendroglioma, Oligoastrocytoma, and Astrocytoma), laterality (Lift, Midline, and Right), molecular subtype (IDHmut−non−codel, IDHmut-codel, and IDHwt).

The RCircos package in R was used to draw a circle plot of the CNV difference between normal and LGG. The distribution of CNV was compared with the Kruskal–Wallis test. The difference (Normal vs. LGG) and heatmap view of the whole methylation landscape of the 17 regulators in LGG were retrieved from DiseaseMeth version 2.0 [9].

### Cluster analysis and differential analysis

An ensemble approach was used to improve predictability and increase robustness. To explorer the specific information with m6A that enabled more detailed decisions in unsupervised class discovery, the samples were clustered into different groups using the ConsensusClusterPlus package in R [10]. Consensus matrix plots of each given number of groups (k, from 2 to 10) depict consensus values transition white to a blue color scale, are sorted by the consensus clustering. Heatmaps were produced depending on the average linkage methodology and the Pearson distance measurement protocol. Principal Component Analysis (PCA) was employed by the R package named PCA to illustrate the clustering conditions of given samples with tumor by dimension reduction. Subsequently, the Limma package (the “lmFit” algorithm with the default parameters) in R was utilized to analyze the different genes between the two clusters. Log2 Fold change (logFC) > 2 and adjusted p-value < 0.01 were set as the cutoff values to screen for differentially expressed genes (DEGs).

### Functional enrichment analysis

The Gene Ontology (GO) analyses and Kyoto Encyclopedia of Genes and Genomes (KEGG) pathway analyses were conducted to annotate the function of up-regulated DEGs using the clusterProfiler package in R [10]. The terms of GO and KEGG with q-value < 0.05 were filtered as significant functions. The Gene Set Enrichment Analysis (GSEA) is a computational protocol utilized to examine whether a set of predefined genes possess statistically significant and consistent deviation between two phenotypes. In this study, GSEA (default parameters) was used to investigate the functions correlated with different clusters of LGG. |NES|>1, adjusted p-value < 0.05 and FDR q value < 0.25 were considered as statistically significant described as previous study [2].

### Identification of potential compounds

To determine which target drugs might be useful against cancer stem cells, we used the Broad Institute’s Connectivity Map build 02 (CM), a public online tool (https://portals.broadinstitute.org/cmap/) (with registration) that allows users to predict compounds that can activate or inhibit based on a gene expression signature. To further investigate about mechanism of actions (MoA) and drug-target we performed specific analysis within Connectivity Map tools (https://clue.io/) [2]. CMap (updated in September 2017) (https://clue.io/), as the world”s largest resource that illuminated the relationships between diseases, cell physiology, and therapeutics based on the transcriptional expression data, was employed to retrieve the potential compounds that specifically target m6A-related pathways in LGG [11]. The candidate compounds (specific targeted molecular drugs) were filtered by comparing the similarity to all perturbational signatures in the database with a list of designed DEGs that relevant to the biological features of interest. A total of 300 DEGs (top 150 downregulated and top150 up-regulated, ordered by logFC values) were considered as “signatures” that arise from different analyses of LGG in different clusters. Afterward, the final compound query matrix involved a connectivity score (tau, ranging from -1 to 1) that indicated the degree of the similarity to the perturbagen than the current query. Notably, the closer the absolute value of tau of a compound is to -1, the more likely it is to be considered to target the gene we are querying (i.e., targeting m6A-related genes). Finally, given the redundancy within the reference panel, compounds with tau in the top 5% were identified as potential therapeutic agents for further study.

### Significance of the MASig in drug sensitivity

To evaluate MASig in the clinic for glioma treatment, the IC50 of commonly administered chemotherapeutic drugs in the TCGA glioma dataset was calculated using the algorithm developed by Geeleher et al. and the corresponding R package ‘pRRophetic’[12, 13]. The algorithm allows users to predict the clinical chemotherapeutic response using only baseline tumor gene expression data, which is achieved by creating statisticalmodels from the gene expression and drug sensitivity data from cell lines in the Cancer Genome Project. The difference in the IC50s of common antitumor drugs between the high and low MASig groups was compared using the Wilcoxon signed-rank test.

Briefly, microarray probes are (when possible) first remapped to the latest build of EntrezID. Training and test expression data are quantile normalized separately and subsequently combined by standardizing the mean and variance of each gene using an empirical Bayesian approach. Genes with very low variability across samples are removed. A ridge regression model is fit to the training expression data using all remaining genes as predictors and the drug sensitivity (IC50) values (of the drug of interest) as the outcome variable. Finally, this model is applied to the processed, standardized, filtered clinical tumor expression data, yielding a drug sensitivity estimate for each patient.

### weighted co-expression network analysis (WGCNA) and module preservation

The WGCNA corresponds to a data reduction method and unsupervised classification method. It simplifies the interpretation of thousands of gene responses to a dozen of synthetic groups (or modules) of genes. The net establishes connections between genes-genes are connected if their expression is correlated. Genes can be more or less intensively connected depending on the value of the correlation (the weights). The connectivity between genes is then interpreted into a distance, and the distance is used to group genes into modules. This is how a high number of genes can be reduced into a small number of clusters whose expression is quantified by the Eigengenes (first principal component within the module). It relies on the assumption that highly correlated genes within a module are involved in common biological processes. The R package named “WGCNA” was used to further analyses and find gene co-expression modules to explore the association between the gene co-network and the phenotype of interest, as well as the key genes in the network [14].

Considering that there is no significant difference between some genes in normal and tumor tissues, to more accurately explore the various role of MAGs in LGG, DEGs (P < 0.05 and logFC > 2) were utilized in subsequent WGCNA analysis to ensure heterogeneity and accuracy. Briefly, RNA-seq data and samples with LGG were filtered to remove the outliers. The weighted network was constructed to calculate the adjacency using the optimal soft thresholding power parameter based on the scale-free topology criteria. The “TOMSimilarity” function was employed to transform the adjacency matrix into a topological overlap matrix (TOM), which measures the network connectivity of genes. The dynamic dendrogram was plotted through the “dynamicMods” function with minModuleSize = 30. After that, for tree dendrograms pruning to obtain co-expression modules, correlated modules (MEDissThres = 0.25) were then merged.

To further explore the association between clinical parameters and module Eigengenes (MEs) in each module. The overlaps of parameters and merged modules were designed with the hypergeometric test, and then the P-value was defined as module membership (MM). Gene significance (GS) was assigned as the correlation between the parameters and the expression pattern of MEs. The hub genes of the most relevant modules were identified with the threshold MM > 0.5 and GS > 0.5.

### Confirmation of significant modules

To further explore the relationship between the clinical phenotypes and designated modules. The co-relationship of and merging modules were measured with the hypergeometric test, and the P-value was assigned as module membership (MM). A table of the P-values with color-coded and phenotypes of interest was presented. Gene significance (GS) was appointed as the correlation between the merged modules and the phenotype.

### Single-Sample Gene-Set Enrichment Analysis (ssGSEA)

ssGSEA was employed to quantify the relative enrichment of each immune cell fraction in the TME with the gene sets from Charoentong’s study [15, 16]. The expression levels of each gene were z-score normalized across all patients. For each patient (or group of patients) genes were then ranked in descending order according to their z-scores (mean of z-scores). The association was represented by a normalized enrichment score (NES). An immune cell type was considered enriched in a patient or group of patients when FDR (q-value) ≤ 10%. Importantly, of the immune cells (ActCD4, ActCD8, TcmCD4, TcmCD8, TemCD4, TemCD8, Th1, Th17, ActDC, CD56briNK, NK, NKT) were defined as anti-tumor immune cells. Treg, Th2, CD56dimNK, imDC, TAM, MDSC, Neutrophil, and pDC were defined as cells that promote tumors formation and immunosuppression [17].

To discover the underlying mechanism of different subgroups, typical biological processes, including (1) Angiogenesis; (2) antigen processing machinery; (3) CD8 T-effector signature; (4) cell cycle; (5) DNA damage repair; (6) DNA replication; (7) epithelial-mesenchymal transition (EMT) markers including EMT1, EMT2, and EMT3; pan-fibroblast (8) FGFR3-related genes; (9) Immune checkpoint; (10) Mismatch repair; (11) Nucleotide excision repair; (12) TGF-β response signature (Pan-F-TBRS); (13) WNT targets was quantified by ssGSEA with a list of gene sets [18]. Nine gene-sets of oncogenic pathways were also introduced into our analysis to explore the mechanism of regulation on different subclasses [19]. The enrichment scores formulated by ssGSEA were leveraged to demonstrate the abundance of each pathway in each sample.

### Construction of Deep Learning Gene-signature

To investigate the predictions of MAGs on prognosis, Cox proportional hazards (CPH) regression (“survival” package in R) in univariable models was employed to measure the significance between the 17 designed genes and OS. Six genes in which P-values were less than 0.3 were determined as the potential candidates to build a promising predictive model. After that, an L1-penalized (LASSO,1000 repeats) was employed to calculate the formulation of gene-signature. Briefly, the minimum value of lambda was derived from 1,000 cross-validations (“1-se” lambda), which corresponding partial likelihood deviance value was the smallest for the Cox model [20, 21]. At last, the coefficients with regression were confirmed by the “cvfit” function with 1000 repeats. The risk score for the gene-signature was calculated accurately following equation:

Risk score = $\sum_{i=1}^{n} Coefficient* Exp$

where Exp is the transcriptome expression of each designed MAG.

### Measurement of the performance of gene-signature

To show the distribution of gene-signature at a macro level, the heatmaps showing risk scores, disease-free status distributions, and potential gene expression patterns of this gene-signature were presented via pheatmap package in R.

Briefly, the K-M curve was presented to measure the prognosis value of patients with LGG between high- and low-risk scores with the two-sided log-rank test. Receiver operating characteristic (ROC) curve analysis within 1, 3, and 5 years to estimate the performance of the gene-signature with the R packages survivalROC [22].

### Comparisons of Somatic Mutations under Different Levels

With advances in cancer genomics and reduction in costs, wholegenome sequencing (WGS) and whole-exome sequencing (WXS) of large cohorts of cancer samples have become the mainstream way of determining genetic abnormalities associated with cancer. Somatic mutation and CNV profiles were gathered from the TCGA data portal. Somatic mutation data classified according to the mutation annotation format (MAF, verscan2) were analyzed by applying the R package "maftools" [23, 24]. Maftools offers a multitude of analysis and visualization modules while only requiring a single input text file containing somatic variants in MAF format. MAF is a standard tab delimited text file format introduced by TCGA for storing and distributing somatic variants, containing complete somatic landscape of the cohort. Fisher”s exact test was used to identify the differential mutation pattern, and genes with a p-value lower than 0.05 were defined as differentially mutated genes. The co-occurrence and mutually exclusive mutations were identified using the CoMEt algorithm[25]. Cancer genes (driver) were identified from a given MAF file via oncodriveCLUST which was originally implemented in Python [26]. The concept is based on the fact that most of the variants in cancer-causing genes are enriched at a few specific loci (aka hot-spots). This method takes advantage of such positions to identify cancer genes. Mutation sites and types of cancer driver genes in both subgroups were visualized with the lollipopPlot2 algorithm. The mutation panorama (top 30) for both subgroups was visualized with the co-oncoplot algorithm. Drug-gene interactions and gene druggability information was compiled from the Drug Gene Interaction database (http://dgidb.org/) by using drugInteractions algorithm [27]. The enrichment of known oncogenic signaling pathways in TCGA cohorts was calculated and visualized by OncogenicPathways function [19].

1. Liu J, Lichtenberg T, Hoadley KA, Poisson LM, Lazar AJ, Cherniack AD, Kovatich AJ, Benz CC, Levine DA, Lee AV, Omberg L, Wolf DM, Shriver CD, et al. An Integrated TCGA Pan-Cancer Clinical Data Resource to Drive High-Quality Survival Outcome Analytics. Cell. 2018; 173(2):400-416 e411.

2. Subramanian A, Tamayo P, Mootha VK, Mukherjee S, Ebert BL, Gillette MA, Paulovich A, Pomeroy SL, Golub TR, Lander ES and Mesirov JP. Gene set enrichment analysis: a knowledge-based approach for interpreting genome-wide expression profiles. Proc Natl Acad Sci U S A. 2005; 102(43):15545-15550.

3. Fabregat A, Sidiropoulos K, Garapati P, Gillespie M, Hausmann K, Haw R, Jassal B, Jupe S, Korninger F, McKay S, Matthews L, May B, Milacic M, et al. The Reactome pathway Knowledgebase. Nucleic Acids Res. 2016; 44(D1):D481-487.

4. Colwill K, Renewable Protein Binder Working G and Graslund S. A roadmap to generate renewable protein binders to the human proteome. Nat Methods. 2011; 8(7):551-558.

5. Franceschini A, Szklarczyk D, Frankild S, Kuhn M, Simonovic M, Roth A, Lin J, Minguez P, Bork P, von Mering C and Jensen LJ. STRING v9.1: protein-protein interaction networks, with increased coverage and integration. Nucleic Acids Res. 2013; 41(Database issue):D808-815.

6. Shannon P, Markiel A, Ozier O, Baliga NS, Wang JT, Ramage D, Amin N, Schwikowski B and Ideker T. Cytoscape: a software environment for integrated models of biomolecular interaction networks. Genome Res. 2003; 13(11):2498-2504.

7. Lan Q, Liu PY, Haase J, Bell JL, Huttelmaier S and Liu T. The Critical Role of RNA m(6)A Methylation in Cancer. Cancer Res. 2019; 79(7):1285-1292.

8. Chen XY, Zhang J and Zhu JS. The role of m(6)A RNA methylation in human cancer. Mol Cancer. 2019; 18(1):103.

9. Xiong Y, Wei Y, Gu Y, Zhang S, Lyu J, Zhang B, Chen C, Zhu J, Wang Y, Liu H and Zhang Y. DiseaseMeth version 2.0: a major expansion and update of the human disease methylation database. Nucleic Acids Res. 2017; 45(D1):D888-D895.

10. Wilkerson MD and Hayes DN. ConsensusClusterPlus: a class discovery tool with confidence assessments and item tracking. Bioinformatics. 2010; 26(12):1572-1573.

11. Subramanian A, Narayan R, Corsello SM, Peck DD, Natoli TE, Lu X, Gould J, Davis JF, Tubelli AA, Asiedu JK, Lahr DL, Hirschman JE, Liu Z, et al. A Next Generation Connectivity Map: L1000 Platform and the First 1,000,000 Profiles. Cell. 2017; 171(6):1437-1452 e1417.

12. Geeleher P, Cox N and Huang RS. pRRophetic: an R package for prediction of clinical chemotherapeutic response from tumor gene expression levels. PLoS One. 2014; 9(9):e107468.

13. Geeleher P, Cox NJ and Huang RS. Clinical drug response can be predicted using baseline gene expression levels and in vitro drug sensitivity in cell lines. Genome Biol. 2014; 15(3):R47.

14. Langfelder P and Horvath S. WGCNA: an R package for weighted correlation network analysis. BMC Bioinformatics. 2008; 9:559.

15. Charoentong P, Finotello F, Angelova M, Mayer C, Efremova M, Rieder D, Hackl H and Trajanoski Z. Pan-cancer Immunogenomic Analyses Reveal Genotype-Immunophenotype Relationships and Predictors of Response to Checkpoint Blockade. Cell Rep. 2017; 18(1):248-262.

16. Hanzelmann S, Castelo R and Guinney J. GSVA: gene set variation analysis for microarray and RNA-seq data. BMC Bioinformatics. 2013; 14:7.

17. Jia Q, Wu W, Wang Y, Alexander PB, Sun C, Gong Z, Cheng JN, Sun H, Guan Y, Xia X, Yang L, Yi X, Wan YY, et al. Local mutational diversity drives intratumoral immune heterogeneity in non-small cell lung cancer. Nat Commun. 2018; 9(1):5361.

18. Mariathasan S, Turley SJ, Nickles D, Castiglioni A, Yuen K, Wang Y, Kadel EE, III, Koeppen H, Astarita JL, Cubas R, Jhunjhunwala S, Banchereau R, Yang Y, et al. TGFbeta attenuates tumour response to PD-L1 blockade by contributing to exclusion of T cells. Nature. 2018; 554(7693):544-548.

19. Sanchez-Vega F, Mina M, Armenia J, Chatila WK, Luna A, La KC, Dimitriadoy S, Liu DL, Kantheti HS, Saghafinia S, Chakravarty D, Daian F, Gao Q, et al. Oncogenic Signaling Pathways in The Cancer Genome Atlas. Cell. 2018; 173(2):321-337 e310.

20. Zhang Y, Li H, Zhang W, Che Y, Bai W and Huang G. LASSObased CoxPH model identifies an 11lncRNA signature for prognosis prediction in gastric cancer. Mol Med Rep. 2018; 18(6):5579-5593.

21. Goeman JJ. L1 penalized estimation in the Cox proportional hazards model. Biom J. 2010; 52(1):70-84.

22. Harrell FE, Jr., Lee KL and Mark DB. Multivariable prognostic models: issues in developing models, evaluating assumptions and adequacy, and measuring and reducing errors. Stat Med. 1996; 15(4):361-387.

23. Koboldt DC, Zhang Q, Larson DE, Shen D, McLellan MD, Lin L, Miller CA, Mardis ER, Ding L and Wilson RK. VarScan 2: somatic mutation and copy number alteration discovery in cancer by exome sequencing. Genome Res. 2012; 22(3):568-576.

24. Mayakonda A, Lin DC, Assenov Y, Plass C and Koeffler HP. Maftools: efficient and comprehensive analysis of somatic variants in cancer. Genome Res. 2018; 28(11):1747-1756.

25. Leiserson MD, Wu HT, Vandin F and Raphael BJ. CoMEt: a statistical approach to identify combinations of mutually exclusive alterations in cancer. Genome Biol. 2015; 16:160.

26. Tamborero D, Gonzalez-Perez A and Lopez-Bigas N. OncodriveCLUST: exploiting the positional clustering of somatic mutations to identify cancer genes. Bioinformatics. 2013; 29(18):2238-2244.

27. Griffith M, Griffith OL, Coffman AC, Weible JV, McMichael JF, Spies NC, Koval J, Das I, Callaway MB, Eldred JM, Miller CA, Subramanian J, Govindan R, et al. DGIdb: mining the druggable genome. Nat Methods. 2013; 10(12):1209-1210.
