# Supplementary material for: Identification of Implications of Angiogenesis and m6A Modification on Immunosuppression and Therapeutic Sensitivity in Low-Grade Glioma by Network Computational Analysis of Subtypes and Signatures
Source: Front Immunol. 2022 Apr 27;13:871564. doi: 10.3389/fimmu.2022.871564 (PMC9094412; doi:10.3389/fimmu.2022.871564)
Supplement: Supplementary file 4 [file Table_2.docx]

| compounds | p-value | compounds | p-value |
| --- | --- | --- | --- |
| Cyclopamine | 1.20E-41 | CHIR.99021 | 6.07E-18 |
| NVP.BEZ235 | 3.23E-41 | Etoposide | 2.21E-17 |
| Pazopanib | 1.41E-40 | Z.LLNle.CHO | 5.77E-17 |
| XMD8.85 | 3.45E-38 | GW843682X | 8.52E-17 |
| FTI.277 | 9.02E-38 | Mitomycin.C | 1.56E-16 |
| Bexarotene | 1.84E-36 | CCT018159 | 2.22E-15 |
| Bryostatin.1 | 3.15E-36 | Gemcitabine | 4.20E-15 |
| A.443654 | 5.79E-34 | MS.275 | 1.10E-14 |
| Bortezomib | 7.54E-34 | Erlotinib | 3.12E-14 |
| Roscovitine | 7.50E-33 | KU.55933 | 2.71E-13 |
| GSK269962A | 1.59E-32 | CMK | 3.54E-13 |
| JW.7.52.1 | 3.22E-32 | BMS.536924 | 5.28E-13 |
| Rapamycin | 3.29E-32 | Vinblastine | 1.94E-12 |
| Bicalutamide | 3.99E-31 | RO.3306 | 3.55E-12 |
| BI.2536 | 7.50E-31 | NSC.87877 | 3.67E-12 |
| NVP.TAE684 | 8.30E-30 | S.Trityl.L.cysteine | 5.21E-12 |
| Dasatinib | 1.00E-29 | AKT.inhibitor.VIII | 6.67E-12 |
| AS601245 | 1.10E-29 | PF.562271 | 1.38E-11 |
| X17.AAG | 2.04E-29 | SB.216763 | 2.70E-11 |
| Embelin | 2.71E-29 | VX.680 | 2.96E-11 |
| Parthenolide | 3.26E-29 | Cytarabine | 1.52E-10 |
| A.770041 | 1.55E-28 | BI.D1870 | 1.24E-09 |
| Salubrinal | 2.29E-28 | AZD6244 | 4.12E-09 |
| GNF.2 | 3.58E-27 | Metformin | 1.20E-08 |
| Tipifarnib | 3.04E-26 | Bleomycin | 1.55E-08 |
| Midostaurin | 6.20E-26 | PF.4708671 | 1.78E-07 |
| PHA.665752 | 9.37E-26 | Elesclomol | 3.50E-07 |
| WZ.1.84 | 2.15E-25 | Sunitinib | 4.34E-07 |
| RDEA119 | 3.80E-25 | BX.795 | 7.17E-07 |
| AP.24534 | 5.86E-25 | Vinorelbine | 1.29E-06 |
| CGP.60474 | 1.09E-24 | JNK.9L | 2.26E-06 |
| MG.132 | 1.61E-24 | GSK.650394 | 3.34E-06 |
| Obatoclax.Mesylate | 1.40E-22 | Temsirolimus | 4.74E-06 |
| Shikonin | 7.00E-22 | Lapatinib | 6.09E-06 |
| AUY922 | 7.73E-22 | AZD.0530 | 6.33E-06 |
| FH535 | 1.82E-21 | CEP.701 | 2.40E-05 |
| AZD6482 | 4.97E-21 | MK.2206 | 2.75E-05 |
| Sorafenib | 6.67E-21 | Pyrimethamine | 3.26E-05 |
| Paclitaxel | 2.00E-20 | Doxorubicin | 6.07E-05 |
| CGP.082996 | 9.07E-20 | Thapsigargin | 9.38E-05 |
| PD.0325901 | 5.03E-19 | LFM.A13 | 0.000295 |
| Docetaxel | 6.46E-19 | Epothilone.B | 0.000478 |
| BMS.509744 | 2.09E-18 | PF.02341066 | 0.002725 |
| Cisplatin | 2.49E-18 | PLX4720 | 0.003454 |
| AZ628 | 3.28E-18 | AZD7762 | 0.006821 |
| WH.4.023 | 4.58E-18 | GDC.0449 | 0.009882 |
| CI.1040 | 0.019613 | BMS.754807 | 0.800273 |
| PD.173074 | 0.044306 | AZD.2281 | 0.846725 |
| Camptothecin | 0.129855 | IPA.3 | 0.963484 |
| Imatinib | 0.156172 | QS11 | 0.969088 |
| JNJ.26854165 | 0.274142 | PD.0332991 | 0.989751 |
| NU.7441 | 0.370297 | KIN001.135 | 0.996554 |
| DMOG | 0.376056 | Methotrexate | 0.999251 |
| TW.37 | 0.464165 | AG.014699 | 0.99934 |
| WO2009093972 | 0.719784 | BAY.61.3606 | 0.999885 |
| GDC0941 | 0.783934 | PAC.1 | 0.999915 |
| AZD8055 | 0.790342 | Vorinostat | 0.999994 |
