# Supplementary material for: Identification of Implications of Angiogenesis and m6A Modification on Immunosuppression and Therapeutic Sensitivity in Low-Grade Glioma by Network Computational Analysis of Subtypes and Signatures
Source: Front Immunol. 2022 Apr 27;13:871564. doi: 10.3389/fimmu.2022.871564 (PMC9094412; doi:10.3389/fimmu.2022.871564)
Supplement: Supplementary file 6 [file Table_4.docx]

| Name | ID | Score | Type | Name | Description |
| --- | --- | --- | --- | --- | --- |
| M2-PK-activator | BRD-K80672993 | -91.94 | cp | M2-PK-activator |  |
| latrunculin-b | BRD-A19248578 | 93.01 | cp | latrunculin-b | Actin polymerization inhibitor |
| naftopidil | BRD-A01787639 | -91.74 | cp | naftopidil | Adrenergic receptor antagonist |
| bufalin | BRD-K63606607 | -92.57 | cp | bufalin | ATPase inhibitor |
| maraviroc | BRD-A04352665 | -95.95 | cp | maraviroc | CC chemokine receptor antagonist |
| PHA-793887 | BRD-K64800655 | 92.95 | cp | PHA-793887 | CDK inhibitor |
| deferiprone | BRD-K06878038 | -94.61 | cp | deferiprone | Chelating agent |
| mycophenolate-mofetil | BRD-K92428153 | 92.42 | cp | mycophenolate-mofetil | Dehydrogenase inhibitor |
| azacitidine | BRD-K03406345 | -90.74 | cp | azacitidine | DNA methyltransferase inhibitor |
| WZ-3146 | BRD-K73293050 | 93.94 | cp | WZ-3146 | EGFR inhibitor |
| toremifene | BRD-K67174588 | -93.95 | cp | toremifene | Estrogen receptor antagonist |
| 7b-cis | BRD-K61829047 | 91.17 | cp | 7b-cis | Exportin antagonist |
| TG-101348 | BRD-K12502280 | 95.28 | cp | TG-101348 | FLT3 inhibitor |
| GANT-58 | BRD-K64451768 | -92.4 | cp | GANT-58 | GLI antagonist |
| memantine | BRD-A79803969 | -91.08 | cp | memantine | Glutamate receptor antagonist |
| TWS-119 | BRD-K94176593 | 92.6 | cp | TWS-119 | Glycogen synthase kinase inhibitor |
| givinostat | BRD-K13810148 | 98.94 | cp | givinostat | HDAC inhibitor |
| ISOX | BRD-K69840642 | 98.94 | cp | ISOX | HDAC inhibitor |
| trichostatin-a | BRD-K68202742 | 98.91 | cp | trichostatin-a | HDAC inhibitor |
| pyroxamide | BRD-K11663430 | 98.91 | cp | pyroxamide | HDAC inhibitor |
| THM-I-94 | BRD-K12867552 | 98.91 | cp | THM-I-94 | HDAC inhibitor |
| scriptaid | BRD-K22503835 | 98.91 | cp | scriptaid | HDAC inhibitor |
| NCH-51 | BRD-K52522949 | 98.87 | cp | NCH-51 | HDAC inhibitor |
| belinostat | BRD-K17743125 | 98.87 | cp | belinostat | HDAC inhibitor |
| vorinostat | BRD-K81418486 | 98.87 | cp | vorinostat | HDAC inhibitor |
| HC-toxin | BRD-A39646320 | 98.84 | cp | HC-toxin | HDAC inhibitor |
| dacinostat | BRD-K56957086 | 98.84 | cp | dacinostat | HDAC inhibitor |
| WT-171 | BRD-K74761218 | 98.8 | cp | WT-171 | HDAC inhibitor |
| apicidin | BRD-K64606589 | 98.34 | cp | apicidin | HDAC inhibitor |
| APHA-compound-8 | BRD-K74733595 | 98.24 | cp | APHA-compound-8 | HDAC inhibitor |
| panobinostat | BRD-K02130563 | 97.22 | cp | panobinostat | HDAC inhibitor |
| geldanamycin | BRD-A19500257 | 95.24 | cp | geldanamycin | HSP inhibitor |
| NVP-AUY922 | BRD-K41859756 | 92.27 | cp | NVP-AUY922 | HSP inhibitor |
| BMS-536924 | BRD-K34581968 | 90.48 | cp | BMS-536924 | IGF-1 inhibitor |
| hydroxycholesterol | BRD-A36707673 | -94.63 | cp | hydroxycholesterol | LXR agonist |
| selumetinib | BRD-K57080016 | 94.06 | cp | selumetinib | MEK inhibitor |
| U-0126 | BRD-K18787491 | 93.26 | cp | U-0126 | MEK inhibitor |
| L-152804 | BRD-A42553870 | -90.94 | cp | L-152804 | Neuropeptide receptor antagonist |
| 3-amino-benzamide | BRD-K08703257 | -92.53 | cp | 3-amino-benzamide | PARP inhibitor |
| PKCbeta-inhibitor | BRD-K89687904 | -96.8 | cp | PKCbeta-inhibitor | PKC inhibitor |
| calyculin | BRD-A47513740 | -97.78 | cp | calyculin | Protein phosphatase inhibitor |
| cycloheximide | BRD-A62184259 | -95.81 | cp | cycloheximide | Protein synthesis inhibitor |
| tyrphostin-AG-112 | BRD-K01192156 | -91.71 | cp | tyrphostin-AG-112 | Protein tyrosine kinase inhibitor |
| triptolide | BRD-A13122391 | 96.44 | cp | triptolide | RNA polymerase inhibitor |
| AGK-2 | BRD-K32536677 | -99.26 | cp | AGK-2 | SIRT inhibitor |
| MST-312 | BRD-K19894101 | 97.5 | cp | MST-312 | Telomerase inhibitor |
| U-46619 | BRD-K18757346 | -92.85 | cp | U-46619 | Thromboxane receptor agonist |
| hinokitiol | BRD-K37691127 | 91.53 | cp | hinokitiol | Tyrosinase inhibitor |
